# Glycaemic responses to metformin monotherapy by SNP clusters in patients with type 2 diabetes

**DOI:** 10.1111/dom.70023

**Published:** 2025-08-22

**Authors:** Wayne Huey‐Herng Sheu, Chun‐Yi Lee, Yi‐Wen Wang, Cai‐Sian Liao, Tzu‐Hung Hsiao, Ken Suzuki, Konstantinos Hatzikotoulas, Andrew P. Morris, the Type 2 Diabetes Global Genetics Initiative, Yii‐Der Ida Chen, Jerome I. Rotter

**Affiliations:** ^1^ Institute of Molecular and Genomic Medicine National Health Research Institutes Zhunan Taiwan; ^2^ Division of Endocrinology and Metabolism, Department of Internal Medicine Taichung Veterans General Hospital Taichung Taiwan; ^3^ Division of Endocrinology and Metabolism, Department of Internal Medicine Taipei Veterans General Hospital Taipei Taiwan; ^4^ Department of Internal Medicine Central Clinic and Hospital Taipei Taiwan; ^5^ College of Medicine National Defense Medical University Taipei Taiwan; ^6^ Bioinformatics Program, Institute of Statistical Science Taiwan International Graduate Program, Academia Sinica Taipei Taiwan; ^7^ Department of Medical Research Taichung Veterans General Hospital Taichung Taiwan; ^8^ Department of Public Health Fu Jen Catholic University New Taipei City Taiwan; ^9^ Institute of Genomics and Bioinformatics National Chung Hsing University Taichung Taiwan; ^10^ Department of Diabetes and Metabolic Diseases, Graduate School of Medicine University of Tokyo Tokyo Japan; ^11^ Institute of Translational Genomics Helmholtz Zentrum München, German Research Center for Environmental Health Neuherberg Germany; ^12^ Centre for Genetics and Genomics Versus Arthritis, Centre for Musculoskeletal Research The University of Manchester Manchester UK; ^13^ The Institute for Translational Genomics and Population Sciences, Department of Pediatrics The Lundquist Institute for Biomedical Innovation at Harbor‐UCLA Medical Center Torrance California USA

**Keywords:** cluster, metformin, SNPs, type 2 diabetes

## Abstract

**Aims:**

This study retrospectively investigates the association between polygenic risk scores (PRS) derived from SNP clusters and glycaemic response to metformin in patients with newly diagnosed T2D.

**Materials and Methods:**

Utilizing a dataset from the Taiwan Precision Medicine Initiative, we evaluated alterations in fasting glucose (FBG) and glycated haemoglobin (HbA1c) in individuals newly diagnosed with T2D who underwent metformin monotherapy for a duration of 6 months. Glycaemic responses between those in the bottom 20% of PRS (Q1) and the top 20% of PRS (Q5) for each of the SNP clusters and for the combination of two clusters were analysed.

**Results:**

In responses to metformin monotherapy, significant differences of FBG levels were detected in Q1 as compared to Q5 in individuals of PRS derived from the cluster of beta‐cell dysfunction with a positive association with proinsulin (Beta cell +PI) (*p* = 0.005) and the cluster of beta‐cell dysfunction with a negative association with proinsulin (Beta cell −PI) (*p* = 0.003). Moreover, lower FBG levels on treatment were observed in those with both Q1 than those with both Q5 in the PRS derived from the two clusters of beta cell dysfunction (*p* = 0.002). Significantly reduced HbA1c values were documented in the Q1 in comparison to the Q5 within the cluster of Beta cell −PI (*p* = 0.002).

**Conclusion:**

These findings suggest that PRS derived from beta‐cell dysfunction clusters may help predict glycaemic response to metformin and support the potential for genetically guided treatment in T2D.

## 1 INTRODUCTION

Globally, the number of people living with type 2 diabetes has been growing significantly over the past decades,^1^ driven by many causes such as ageing populations and rising prevalence of obesity.^2^ Despite multiple interventions and the introduction of novel treatment regimens and medications, a significant proportion of individuals with diabetes continue to experience suboptimal glycaemic control,^3^, ^4^, ^5^ leading to substantial healthcare burdens and expenses.

Metformin, a widely prescribed oral medication, primarily lowers blood glucose by inhibiting hepatic gluconeogenesis and improving insulin sensitivity, mainly through activation of AMP‐activated protein kinase and mitochondrial effects, is considered the first‐line treatment for patients with type 2 diabetes. Presently, most national, regional and international guidelines endorse metformin as the primary initial therapeutic option^6^ unless contraindications are present. Nevertheless, the glycaemic response to metformin therapy exhibits considerable variability among patients, with some attaining exceptional glycaemic control, while others display minimal or no therapeutic efficacy. The heritability of the alterations in glycated haemoglobin (HbA1c) in response to metformin therapy has been estimated to be approximately 34%, indicating that a substantial fraction of the treatment response is influenced by genetic factors.^7^

Genetic determinants of response to metformin therapy in diabetes have been investigated through genome‐wide association studies (GWAS) and targeted exome and related pathophysiology pathways. A GWAS conducted within the Diabetes Prevention Program revealed that specific ancestry‐specific variants located near the *ENOSF‐1* region were correlated with variations in percentage glycated haemoglobin, while variants in proximity to *OMSR* were linked to weight loss in individuals undergoing metformin treatment.^8^ Another investigation identified genetic variants at rs1050152 and rs272893 within the *SLC22A4* gene, which were associated with enhanced responses to metformin in treatment‐naïve diabetic individuals, whereas non‐responders exhibited a loss of copy number in the *PPARGC1A* gene.^9^ Recently, Wu et al.^10^ identified a specific variant, rs143276236, within the *ARFGEF3* gene, which was associated with the glycaemic effects of metformin in African American individuals with type 2 diabetes. Although findings in the latter study remained significant in the subsequent meta‐analysis, none of the significant discovery variants were replicated in participants of European American descent in the DIAMOND study. Collectively, these findings underscore the intricate interactions of genetic variations that influence the differential glycaemic responses to metformin treatment.

Both prevalence and incidence of type 2 diabetes are rising in Taiwan.^11^ The pathophysiology and characteristics of type 2 diabetes, as reported from other countries in East Asia, usually present at a lower mean BMI, early beta‐cell dysfunction and lower insulin resistance as compared with those of European descent. These unique pathophysiologies may contribute to a higher prevalence of diabetes at a younger age and renal complications.^12^, ^13^, ^14^

Recently, Suzuki and collaborators in the largest multi‐ancestry GWAS of T2D to date^15^ identified 1289 independent single nucleotide polymorphisms (SNPs), which they were then able to categorise into eight distinct clusters at 611 loci. This significant finding was achieved through the analysis of aggregated data derived from meta‐analysis of cohorts exceeding 2.5 million participants, which included 428 452 cases of type 2 diabetes, encompassing diverse ancestry groups, with approximately 20% of participants being of East Asian ancestry. The eight unique non‐overlapping clusters of SNPs not only demonstrated differential enrichment across various cell types, but also revealed specific profiles of associations with cardiometabolic traits, and highlighted that partitioned polygenic risk score (PRS) was associated with vascular outcomes. Nevertheless, the potential association of these risk alleles within each cluster from Suzuki et al.^15^ and glycaemic response to metformin monotherapy has not been previously investigated. The primary aim of this research was to examine the glycaemic response to metformin monotherapy by employing the dataset procured from a subset of the Taiwan Precision Medicine Initiative (TPMI), concentrating on individuals of Han Chinese descent diagnosed with type 2 diabetes in Taiwan.

## 2 MATERIALS AND METHODS

### 2.1 Study population

Study population data were obtained from the TPMI, which is a collaboration between several major hospitals nationwide and Academia Sinica. This initiative focuses on utilising advanced technologies and data analysis to tailor medical treatments to individual patients, thereby improving the effectiveness of healthcare delivery.^16^ The goal of TPMI was to incorporate genetic information into clinical implementations. (Available online from https://tpmi.ibms.sinica.edu.tw/) (Accessed April 27, 2025). Blood samples of each participant enrolled in the TPMI were collected, DNA was extracted and genotyped (see below). The genetic profiles of TPMI participants are linked to their electronic health records 5 years before and 3 years after enrollment. Between June 2019 and May 2021, a total of 57 257 hospital outpatient participants were enrolled at the Taichung Veterans General Hospital (Taichung VGH) site of the TPMI project. All participants signed written consent before commencement of all interviews and examinations.

**FIGURE 1:**
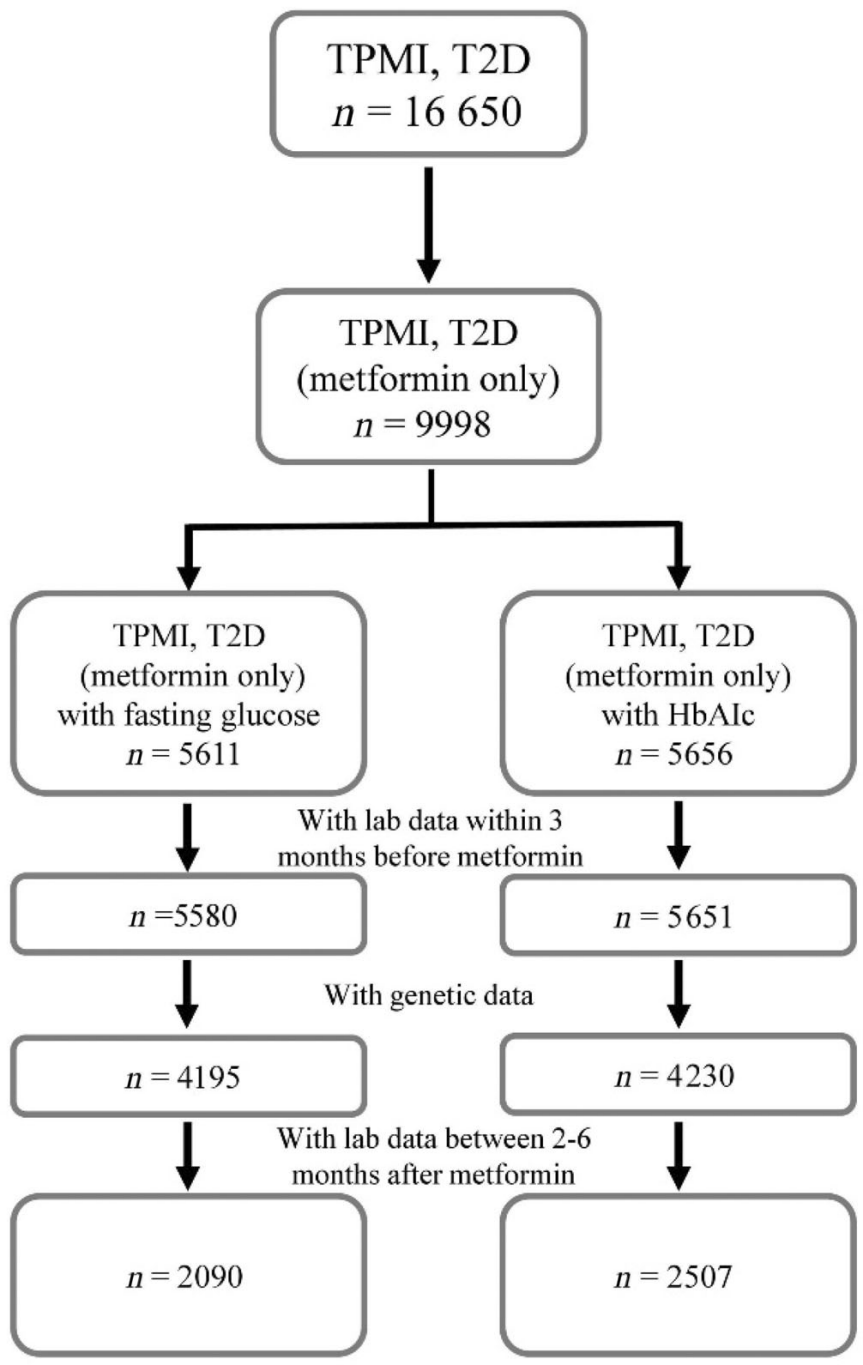
Flow chart of study design.

We identified 16 650 individuals with a diagnosis of type 2 diabetes based on the International Classification of Diseases, Ninth/Tenth Revision and Clinical Modifications (ICD‐9/10 CM).^17^ Individuals with a diagnosis of type 1 diabetes, gestational diabetes mellitus alone, or drug‐associated diabetes (e.g., because of corticosteroid use) were excluded. Subsequently, we filtered based on the availability of lab data taken within 3 months prior to starting metformin monotherapy. This left 5580 patients in the FBG group and 5651 patients in the HbA1c group. Additionally, genotypic data were available for a subset of patients, as not all participated in the TPMI, reducing the sample sizes to 4195 and 4230 for FBG and HbA1c groups, respectively. The final analysis focused on those with multiple follow‐up lab data between 2 and 6 months after initiating metformin therapy, resulting in two final cohorts: 2090 patients in the FBG group and 2,507 in the HbA1c group (*n* = 1853 of them were present in both groups). This stepwise filtering and stratification ensure a retrospective cohort study design aiming to assess changes in FBG and HbA1c levels after metformin administration, potentially influenced by genetic factors (Figure 1).

### 2.2 Genotyping

DNA extraction and genotyping were performed on automated platforms at the research lab at Taichung Veterans General Hospital, Taiwan. Genotyping of each participant was performed using Taiwan Biobank version 2 (TWBv2) array (Thermo Fisher Scientific, Inc., Santa Clara, CA, USA), which was designed in 2017 and tested with a total of 714 431 SNPs, as previously described by Wei and colleagues.^18^ To maximise accuracy and prevent batch effects, Academia Sinica conducted genotype calls centrally for batches of 3000 samples each.

SNPs with missing rate greater than 0.05, or minor allele frequency less than 0.05, or failing the Hardy–Weinberg equilibrium test (*p* < 1E‐05) were excluded.^19^ Array data were imputed on Michigan imputation server^20^ using 1000 Genomes Phase 3 (Version 5) as reference panel.^21^ All biallelic variants with imputation quality (INFO score) ≥0.3 were included in the analysis.

### 2.3 Calculation of PRS of reported 8 clusters

Eight non‐overlapping clusters of T2D signals were derived from the study by Suzuki et al. which is based on the largest GWAS to date, encompassing 2.5 million individuals.^15^ The eight clusters were defined^15^ as follows, residual glycaemic, obesity, beta‐cell dysfunction with a positive association with proinsulin (Beta cell +PI), beta‐cell dysfunction with a negative association with proinsulin (Beta cell −PI), metabolic syndrome, liver/lipid metabolism, lipodystrophy and body fat. We found 1109 SNPs (86.0%) overlapping with our array data. Table S1 listed the number of SNPs of 8 clusters used in TPMI dataset as compared to those from Suzuki et al.^15^ PLINK v1.90^22^ was applied to calculate cluster‐specific partitioned PRS based on those SNPs of each cluster as ${\mathrm{PRS}}_{i}=\sum_{j=1}^{N_{i}}\beta_{\mathrm{ij}}x_{\mathrm{ij}},$ ^23^ where $N_{i}$ is the number of SNPs of the *i*th cluster, $x_{\mathrm{ij}}$ is the genotype for the *j*th SNP of the *i*th cluster (encodes as 0, 1 or 2 for the additive genetic effect), and $\beta_{\mathrm{ij}}$ is the estimated effect size for the corresponding genotype $x_{\mathrm{ij}}$ obtained from Suzuki et al.^15^ Effect sizes from multi‐ancestry populations were first used, and those from East Asian ancestry were subsequently applied for comparison.^15^ Individuals with type 2 diabetes were divided into five groups equally based on quintiles of each cluster‐specific PRS, denoted as Q1 (0%–19%), Q2 (20%–39%), Q3 (40%–59%), Q4 (60%–79%) and Q5 (80%–100%).

### 2.4 Statistical analysis

Baseline characteristics were analysed by comparing individuals with a dataset of multiple values of FBG and HbA1c levels. The Kruskal‐Wallis and Chi‐square tests were conducted to evaluate the differences between two groups for continuous and dichotomous variables, respectively. Tracking electronic medical records (EMR) from Taichung VGH, we analysed changes in FBG and HbA1c, based on the 8 SNP cluster quintiles, in individuals with type 2 diabetes who received metformin monotherapy. Generalised estimating equations (GEE) were used to compare the glycaemic responses in those with Q5 versus Q1 in each of the SNP clusters or in combination of two clusters adjusted for age and gender. GEE incorporates all repeated measurements from each subject simultaneously, accounting for within‐subject correlations across time points.^24^ Finally, clinical characteristics of individuals carrying Q5 versus Q1 of PRS of Beta cell +PI, Beta cell −PI, residual glycaemic and obesity were also compared using Kruskal‐Wallis and Chi‐square tests. Statistical significance was defined as *p* < 0.05 (*), and Bonferroni‐corrected significance as *p* < 0.05/number of tests (**).

## 3 RESULTS

No significant differences were observed in age, gender distribution, BMI, FBG concentrations or HbA1c levels between the two groups with multiple laboratory examinations of FBG and of HbA1c (Table S2). The reduction in FBG concentrations (mean ± SD: 12.5 ± 27.2 vs. 13.0 ± 26.8 mg/dL) and HbA1c levels (1.1 ± 1.6 vs. 1.1 ± 1.7%) after approximately 6 months of metformin use was comparable between the groups. Daily metformin dosages and duration of metformin use, after Bonferroni's corrections, were similar.

**FIGURE 2:**
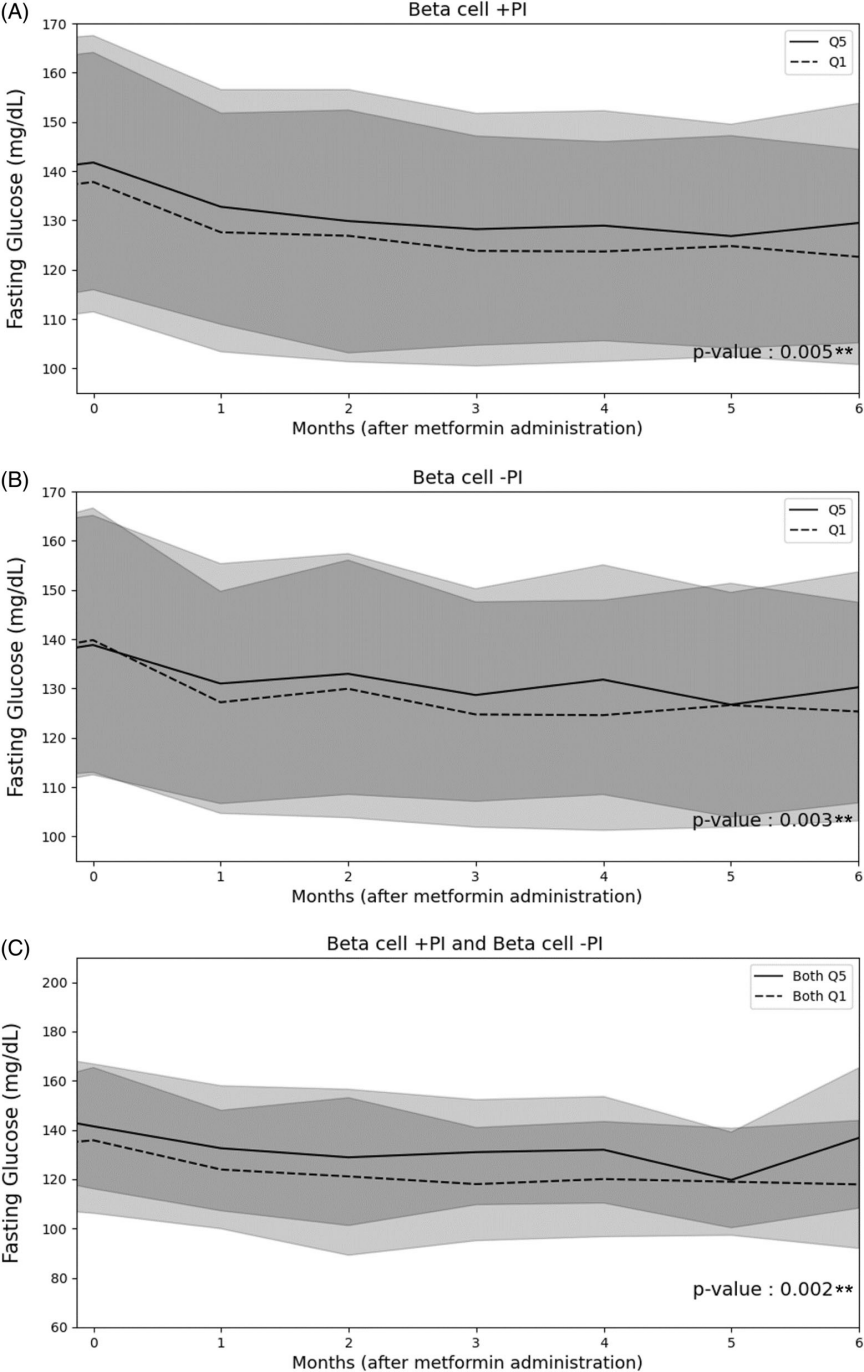
Trends of FBG responses to metformin monotherapy of Q5 vs. Q1 in Beta cell +PI (A**) and Beta cell ‐PI (B**) and combination of two clusters (C**). * denotes nominal significance (*p* < 0.05); ** denotes Bonferroni‐corrected significance. Solid line represents Q5 while dashed line represents Q1. Grey band shows 95% confidence interval. Abbreviations: Beta cell +PI or Beta cell ‐PI, beta‐cell dysfunction with a positive or negative association with proinsulin, respectively. fat (F). Solid line represents Q5 while dashed line represents Q1. Grey band shows 95% confidence interval. Abbreviations: Beta cell +PI, beta‐cell dysfunction with a positive association with proinsulin.

**FIGURE 3:**
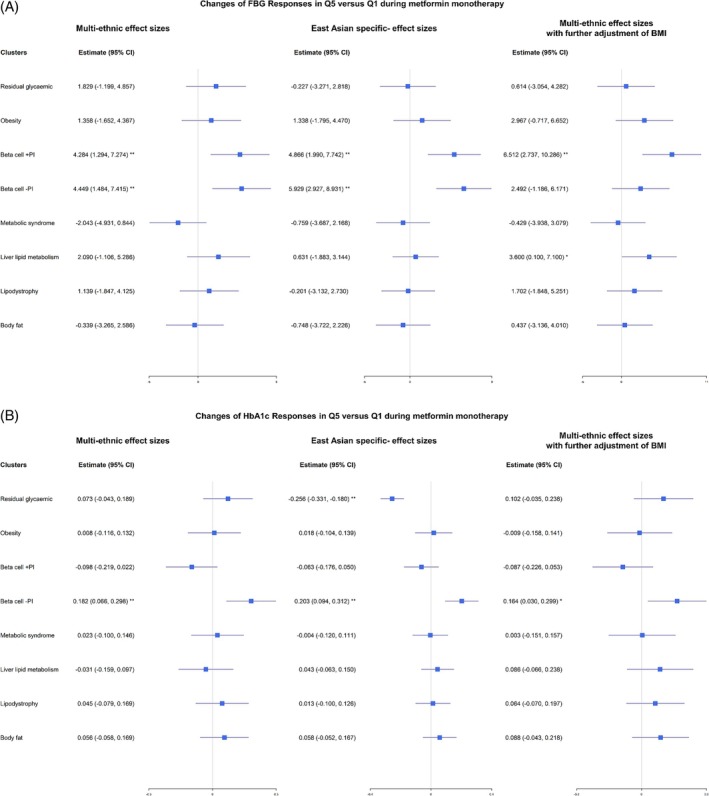
Estimates and 95% CI for the changes of FBG responses (A) and HbA1c responses (B) to metformin monotherapy of Q5 versus Q1 in each cluster with multi‐ethnic effect sizes (left), East Asian specific effect sizes (middle) and multi‐ethnic effect sizes with further adjustment of BMI (right). *Nominal significance (*p* < 0.05); **Bonferroni‐corrected significance (*p* < 0.05/8 = 0.006). Significant differences in FBG levels were detected between Q1 and Q5, especially in Beta cell +PI. Significant differences in HbA1c levels were detected between Q1 and Q5 in Beta cell −PI.

Notably, significant differences in FBG levels were detected between Q1 and Q5 in Beta cell +PI and Beta cell −PI, with beta estimates of 4.284 mg/dL (95% CI: [1.294, 7.274], *p* = 0.005) and 4.449 mg/dL (95% CI: [1.484, 7.415], *p* = 0.003), respectively (Table 1). Throughout the metformin treatment period, persistent significant differences in FBG levels were found between Q1 and Q5 for the partitioned PRS in these two clusters (Figure 2A,B). When testing the combination of the two SNP clusters, significantly lower FBG levels were observed in both Q1 compared to both Q5 in Beta cell +PI and Beta cell −PI, with a beta estimate of 10.653 mg/dL (95% CI: 3.922, 17.385). These differences remained significant when performing multiple testing with Bonferroni's corrections (*p* = 0.002) (Table 1). Trends of persistent differences in FBG were also illustrated in these two clusters (Figure 2C). Trends in FBG values across other clusters, alone or in combination between Q1 versus Q5, during metformin administration are illustrated in Figures S1A–F and S2A–E. Similar results were observed using PRS with East Asian specific‐effect sizes (Table S3). The differences in FBG levels between Q1 and Q5 in Beta cell +PI remained significant with further adjustment of BMI. Combining the two clusters, Beta cell +PI and obesity also showed differences in FBG levels between both Q1 and both Q5 (beta estimate 14.842, 95%CI: [6.380, 23.303], *p* = 0.001) (Table S4). On the other hand, comparable results were found using continuous PRS scores (Table S5). Figure 3A summarises the changes in FBG levels between Q1 and Q5.

**TABLE 1:** Changes of FBG Responses in Q5 versus Q1 during metformin monotherapy by one or two clusters of risk alleles.

Clusters	PRS group	Estimate	95% CI	*p*‐value
Residual glycaemic	Q5 vs. Q1	1.829	(−1.199, 4.857)	0.236
Obesity	Q5 vs. Q1	1.358	(−1.652, 4.367)	0.377
**Beta cell +PI**	**Q5 vs. Q1**	**4.284**	**(1.294, 7.274)**	**0.005** **
**Beta cell −PI**	**Q5 vs. Q1**	**4.449**	**(1.484, 7.415)**	**0.003** **
Metabolic syndrome	Q5 vs. Q1	−2.043	(−4.931, 0.844)	0.165
Liver/lipid metabolism	Q5 vs. Q1	2.090	(−1.106, 5.286)	0.200
Lipodystrophy	Q5 vs. Q1	1.139	(−1.847, 4.125)	0.455
Body fat	Q5 vs. Q1	−0.339	(−3.265, 2.586)	0.820
**Beta cell +PI and Beta cell −PI**	**Both Q5 vs. Both Q1**	**10.653**	**(3.922, 17.385)**	**0.002** **
**Beta cell +PI and residual glycaemic**	**Both Q5 vs. Both Q1**	**8.653**	**(2.417, 14.889)**	**0.007** **
**Beta cell +PI and obesity**	**Both Q5 vs. Both Q1**	**7.441**	**(0.172, 14.709)**	**0.045** *
**Beta cell −PI and residual glycaemic**	**Both Q5 vs. Both Q1**	**9.689**	**(2.745, 16.633)**	**0.006** **
Beta cell −PI and Obesity	Both Q5 vs. Both Q1	5.402	(−1.845, 12.649)	0.144
Residual glycaemic and Obesity	Both Q5 vs. Both Q1	1.653	(−4.883, 8.188)	0.620

Abbreviations: FBG, fasting blood glucose; Beta cell +PI or Beta cell −PI, beta‐cell dysfunction with a positive or negative association with proinsulin (PI), respectively.

* Nominal significance (*p* < 0.05, in bold).

** Bonferroni‐corrected significance (*p* < 0.006 [0.05/8 for single cluster] and *p* < 0.008 [0.05/6, for both Q5 vs. both Q1], in bold).

Turning to HbA1c, significantly lower HbA1c values were found in the Q1 group compared to Q5 in the partitioned PRS derived from the cluster of Beta cell −PI (beta estimate 0.182%, 95% CI: [0.066, 0.298], *p* = 0.002) (Table 2), with persistent significant differences throughout the treatment period (Figure S3A). No differences in HbA1c were noted between Q1 and Q5 in the cluster of residual glycaemic (Figure S3B). Although combining two clusters of Beta cell −PI and residual glycaemic resulted in certain levels of differences in HbA1c (beta estimate 0.290%, 95%CI: [0.030, 0.549], *p* = 0.029), it did not reach stringent threshold of significance by multiple testing with Bonferroni's corrections (Table 2 and Figure S3C). Trends of differences of HbA1c values in other clusters, alone or in combination between Q1 versus Q5 to metformin administration remained insignificances and were illustrated in Figures S4A–F and S5A–E. Consistent results were observed using PRS with East Asian specific‐ effect sizes (Table S6). Further adjustment of BMI did not change our main findings (Table S7). By using continuous PRS scores, similar findings were obtained, namely, only the cluster of Beta cell −PI reached statistical significance after Bonferroni's correction. (Table S8). Figure 3B presents the changes in HbA1c values between Q1 and Q5. In addition, the effect sizes appeared to increase across the PRS spectrum; however, due to the sample sizes, there was certain variation. The effect sizes of Beta cell −PI cluster appeared to show a linear increase across the PRS spectrum (Figure S6).

**TABLE 2:** Changes of HbA1c Responses in Q5 versus Q1 during metformin monotherapy by one or two clusters of risk alleles.

Clusters	PRS group	Estimate	95% CI	*p*‐value
Residual glycaemic	Q5 vs. Q1	0.073	(−0.043, 0.189)	0.219
Obesity	Q5 vs. Q1	0.008	(−0.116, 0.132)	0.902
Beta cell +PI	Q5 vs. Q1	−0.098	(−0.219, 0.022)	0.109
**Beta cell −PI**	**Q5 vs. Q1**	**0.182**	**(0.066, 0.298)**	**0.002** **
Metabolic syndrome	Q5 vs. Q1	0.023	(−0.100, 0.146)	0.713
Liver/lipid metabolism	Q5 vs. Q1	−0.031	(−0.159, 0.097)	0.634
Lipodystrophy	Q5 vs. Q1	0.045	(−0.079, 0.169)	0.475
Body fat	Q5 vs. Q1	0.056	(−0.058, 0.169)	0.336
**Beta cell −PI and Residual glycaemic**	**Both Q5 vs. Both Q1**	**0.290**	**(0.030, 0.549)**	**0.029** *
Beta cell −PI and Obesity	Both Q5 vs. Both Q1	0.008	(−0.281, 0.296)	0.959
Beta cell −PI and Beta cell +PI	Both Q5 vs. Both Q1	0.044	(−0.221, 0.310)	0.743
Beta cell +PI and Residual glycaemic	Both Q5 vs. Both Q1	0.112	(−0.145, 0.368)	0.394
Beta cell +PI and Obesity	Both Q5 vs. Both Q1	0.002	(−0.353, 0.356)	0.993
Residual glycaemic and Obesity	Both Q5 vs. Both Q1	0.112	(−0.191, 0.414)	0.470

Abbreviations: HbA1c, glycated haemoglobin; Beta cell +PI or Beta cell −PI, beta‐cell dysfunction with a positive or negative association with proinsulin (PI), respectively.

* Nominal significance (*p* < 0.05, in bold).

** Bonferroni‐corrected significance (*p* < 0.006 [0.05/8 for single cluster] and *p* < 0.008 [0.05/6, for both Q5 vs. both Q1], in bold).

Overall, clinical features, including age, BMI, FBG concentrations, HbA1c levels and metformin dosages, were generally comparable between individuals in Q1 and Q5 of the partitioned PRS derived from the clusters of Beta cell +PI, Beta cell −PI, residual glycaemic and obesity (Tables S9–S12).

## 4 DISCUSSION

Across eight partitioned clusters of T2D SNPs reported recently,^15^ we found that individuals with type 2 diabetes carrying fewer risk alleles (Q1) of reported Beta cell +PI and Beta cell −PI presented with significantly lower FBG levels as compared to those carrying more risk alleles (Q5) in response to metformin monotherapy for approximately 6 months. Furthermore, individuals carrying fewer risk alleles of those two clusters together (both Q1) showed even greater differences in FBG levels to metformin administration when compared to those carrying more risk alleles (both Q5) with estimated reduction of beta values of 10.653 mg/dL as compared to 4.284 and 4.449 mg/dL in each cluster alone (Table 1). When we examined the differences in HbA1c values, individuals with diabetes carrying fewer reported risk alleles of Beta cell −PI (Q1) presented with lower HbA1c values as compared to those having more risk alleles (Q5) in response to metformin use (Table 2). The combination of a fewer number of reported risk alleles associated with Beta cell −PI and other clusters did not obtain significantly lower HbA1c values compared to individuals harbouring a greater number of reported risk alleles (both Q5) under metformin administration.

Our principal findings indicate that individuals with diabetes who possess the specified SNPs linked to two distinct forms of beta cell dysfunction exhibit a significant and sustained disparity in glucose levels when treated with metformin. These observations are consistent with previous reports that these two clusters were associated with higher FBG, higher 2‐h glucose and higher HbA1c while with decreased fasting insulin.^15^

It is noteworthy that both clusters shared the same features of enhanced insulin sensitivity and decreased insulin secretion while presented with opposite directions of effects of proinsulin on the pathogenesis of type 2 diabetes. Ethnic distinctions in the pathogenesis of T2D have been shown in previous studies that in Europeans, T2D is related to increased insulin resistance, while in East Asians, T2D is characterised by decreased insulin secretion with lower insulin resistance.^13^, ^14^, ^15^

In our assessment of the alterations in HbA1c levels resulting from metformin monotherapy, we discerned that individuals with diabetes who possessed a lesser number of risk alleles from the cluster of Beta cell −PI demonstrated persistent discrepancies in HbA1c values when juxtaposed with those carrying a greater number of risk alleles. In addition, subjects with type 2 diabetes who had fewer risk alleles from both clusters of Beta cell −PI and residual glycaemic (both Q1) exhibited an even more pronounced gap in HbA1c values compared to both quintile 5 classifications (both Q5) despite it not reaching statistical significance by using multiple testing with Bonferroni's corrections. Interestingly, the risk alleles for type 2 diabetes identified at the SNPs attributed to the residual glycaemic cluster—a relatively newly recognised cluster,^25^, ^26^—were reported to encompass the highest quantity of risk alleles among the eight clusters, displaying a robust association with augmented FBG and glycated haemoglobin levels. Recently, it has been shown that metformin upregulated GLP‐1 receptor level and protein kinase A (PKA) phosphorylation.^27^ Metformin treatment might also alter gut microbiota composition, which enhances the production of short‐chain fatty acids (SCFAs) that are linked to increased GLP‐1 secretion.^28^

Although it is well known that the main actions of metformin are on gluconeogenesis and glycolysis, its effects on beta cell remained speculative. Clinically, the use of metformin results in better glycaemic control which in turn lower progression of beta cell failure.^29^ It has been shown that metformin attenuated the depletion of the beta‐cell pool in the streptozotocin‐induced diabetes mice,^30^ perhaps via its anti‐oxidative effects by reducing H_2_O_2_‐induced apoptosis in pancreatic beta‐cells.^31^ In line with our findings that genetic predisposition to beta‐cell dysfunction (with or without proinsulin involvement) modifies response to metformin, a recent report with the individuals of prediabetes from Diabetes Prevention Program, Billings et al.^32^ demonstrated that higher beta‐cell partitioned polygenic score was associated with lowers insulinogenic index and with increased diabetes incidence with adjustment for BMI.

In light of the rapid advancements in population genomics, pharmacogenomic investigations aimed at predicting glycaemic responses to diverse glucose‐lowering pharmacotherapies have been extensively explored. It has been estimated that the heritability of glycaemic response to metformin exhibits variability contingent upon the response phenotype, with a heritability index of 34% for the absolute reduction in HbA1c, adjusted for baseline HbA1c levels.^7^ Prior investigations have assessed the genetic determinants of glycaemic responses to metformin by either focusing on specific variants or on groups of variants localised within particular genes.^8^, ^9^, ^33^, ^34^, ^35^ The current study scrutinised the glycaemic response to metformin across several groups of reported genome‐wide identified clusters of T2D associated SNPs, with a particular emphasis on targeting the underlying pathophysiological pathways pertinent to type 2 diabetes. In support of our study hypothesis, findings from earlier research have indicated that the genetic influence on glycaemic responses to metformin likely arises from individual variants dispersed throughout the genome, each contributing a small to moderate effect, rather than from a limited number of loci that exert a somewhat larger effect.^7^

The efficacy of metformin monotherapy exhibited considerable variability contingent upon factors such as baseline HbA1c levels, lifestyle modifications, potential drug–drug interactions and gut microbiota profiles.^6^ Early meta‐analyses of randomised controlled trials showed that metformin administration resulted in a reduction of fasting plasma glucose levels by 2.0 mmol/L and a decrease in HbA1c by 0.9% relative to placebo.^36^ Our findings indicated a diminished response in FBG levels; however, a modestly enhanced reduction in HbA1c was observed in relation to metformin therapy when compared with a recent investigation involving African American individuals with type 2 diabetes,^10^ who presented with a higher body mass index (BMI), lower initial HbA1c levels and higher metformin dosages compared to participants in our study. These inconsistencies may be attributable to variations in study populations and ethnic backgrounds, as the manifestation of type 2 diabetes in Asian cohorts is characterised by unique phenotypic traits, including elevated postprandial hyperglycemia.^4^, ^37^

It is noteworthy that genetic variants influencing glycaemic responses to metformin may exhibit race‐specific characteristics. For instance, the variant rs143276236 located in the *ARFGEF3* gene has been correlated with metformin's glycaemic responses specifically in African American individuals with type 2 diabetes from the DIAMOND cohort, a finding that has been corroborated in the KNPC African American demographic.^10^ Conversely, none of the significant discovery variants demonstrated replication in the European American participants within the DIAMOND study. Further validation studies across diverse ethnic backgrounds are unequivocally warranted. Recently, Billings et al.^32^ reported that participants of the Diabetes Prevention Program who presented with higher beta‐cell partitioned polygenic scores were associated with reduced beta cell function at baseline and further decline 1 year later despite diabetes prevention interventions, including metformin. The reasons for their findings that no interaction between genetic risk of beta cell dysfunction and metformin might come from ethnic differences and/or preserved certain beta cell function found in prediabetic individuals.^38^

Of note, this is the retrospective data analysis and physicians were not aware of the genotypes of their patients when prescribing metformin. There were certain significant differences in age, BMI and doses of metformin administration between individuals of type 2 diabetes in the Q1 versus those in the Q5 in two clusters pertaining to beta‐cell dysfunction, residual glycaemic and obesity (Tables S9–S12). However, neither FBG levels nor HbA1c values showed significant differences (Tables S9–S12). Our results are not unexpected, as the principal glycaemic response to glucose‐lowering pharmacological agents is widely recognised to be influenced by pretreatment glycaemic levels,^39^, ^40^ rather than the variants associated with susceptibility alleles for type 2 diabetes.

While the primary objective of this study was to furnish clinicians with insights to forecast glycaemic responses to metformin therapy predicated on genetic determinants, the analysis of retrospective data derived from real‐world contexts presents numerous challenges. We concentrated on metformin monotherapy to mitigate any unforeseen implications arising from hypoglycaemic drug–drug interactions. The necessity for laboratory assessments conducted at least once prior to and multiple assessments following the initiation of metformin, alongside the requirement for participants to undergo genetic analysis, further curtailed the number of individuals eligible for the final evaluation. One of the challenges inherent in defining a glycaemic response in individuals diagnosed with type 2 diabetes was the duration of the therapeutic intervention. In light of prior study designs, such as the UKPDS^41^ and GRADE,^42^ it was observed that both studies demonstrated a consistent reduction in HbA1c levels between 6 and 12 months following the initiation of new pharmacotherapy. The current investigation aligns with these findings and has systematically gathered data approximately 6 months subsequent to the commencement of metformin administration. Finally, these hard cluster classifications with each index SNP were assigned to a singular cluster might not meet exactly the underlying pathophysiological pathways.

The limitations included this is real‐world data based on EMRs, and the findings may not be generalisable to other ethnic groups. The current sample size was not sufficient to further investigate other hypoglycaemic drugs. In the future, we plan to expand it in order to examine treatment responses to different regimens of hypoglycaemic drugs—either monotherapy or combination therapy, with or without injectable medications—based on the PRS of different SNP clusters.

In conclusion, our findings elucidated the potential differences in FBG and HbA1c concentrations between individuals possessing fewer or a greater number of risk alleles within the clusters associated with Beta cell +PI and Beta cell −PI to metformin monotherapy over a duration of approximately 6 months. The results derived from the present study offer valuable insights into the genetic determinants that contribute to the variability observed in glycaemic response to metformin, thereby suggesting avenues for personalised treatment strategies in diabetes management based on genetic profiling.

## AUTHOR CONTRIBUTIONS

CYL, YWW and WHHS designed and wrote the paper; CSL and THH performed the statistical analyses; and KS, KH, APQ, JIR, YDI and WHHS conducted the research and contributed to the reviewing and editing of the manuscript.

## CONFLICT OF INTEREST STATEMENT

There are no conflicts of interest from all the authors.

## PEER REVIEW

The peer review history for this article is available at https://www.webofscience.com/api/gateway/wos/peer-review/10.1111/dom.70023.

## Supporting information


**Data S1.** Supporting Information.

## Data Availability

All data we analyzed following the regulation locally and internaionally.
