# Supplementary material for: Glycaemic responses to metformin monotherapy by SNP clusters in patients with type 2 diabetes
Source: Diabetes Obes Metab. 2025 Aug 22;27(11):6323–32. doi: 10.1111/dom.70023 (PMC12515771; doi:10.1111/dom.70023)

Table S1. Number of SNPs in 8 clusters used in present study and reported by Suzuki et al.

| Cluster | Suzuki et al.’s data* | TPMI |
| --- | --- | --- |
| Residual glycaemic | 389 | 339 |
| Obesity | 233 | 212 |
| Beta cell +PI | 91 | 71 |
| Beta cell -PI | 89 | 76 |
| Metabolic syndrome | 166 | 143 |
| Liver/lipid metabolism | 3 | 3 |
| Lipodystrophy | 45 | 38 |
| Body fat | 273 | 227 |
| Total | 1289 | 1109 |

*Suzuki, K., Hatzikotoulas, K., Southam, L. et al. Genetic drivers of heterogeneity in type 2 diabetes pathophysiology. Nature 2024:627, 347–357.

Abbreviations: Beta cell +PI or Beta cell -PI, beta-cell dysfunction with a positive or
negative association with proinsulin, respectively.

Table S2. Baseline characteristics of subjects with type 2 diabetes with dataset of multiple values of fasting glucose and HbA1c levels

| Characteristics | Subjects with multiple  fasting glucose values  (N=2,090) | Subjects with multiple  HbA1c values  (N=2,507) | p-value |
| --- | --- | --- | --- |
| Age (years) | 59.1 ± 11.6 | 59.1 ± 11.7 | 0.907 |
| Female, n (%) | 912 (43.6) | 1120 (44.7) | 0.480 |
| BMI (kg/m^2^) | 26.7 ± 4.0 | 26.6 ± 3.9 | 0.364 |
| Baseline Fasting Glucose (mg/dl) | 138.1 ± 26.5 | 137.8 ± 26.1 | 0.852 |
| Change in Fasting Glucose on metformin treatment | -12.5 ± 27.2 | -13.0 ± 26.8 | 0.655 |
| Baseline HbA1c (%) | 7.8 ± 1.7 | 7.9 ± 1.8 | 0.307 |
| Change in HbA1c on metformin treatment | -1.1 ± 1.6 | -1.1 ± 1.7 | 0.831 |
| Metformin use (mg/day) | 556 ± 226 | 554 ± 221 | 0.995 |
| **Duration of metformin use (month)** | **4.7 ± 1.4** | **4.9 ± 1.3** | **0.037*** |

Data are presented as either the number (percentage) or as the mean ± standard deviation. The Kruskal-Wallis test or$\chi_{2}^{2}$test was conducted to evaluate the differences between two groups.

* denotes nominal significance (*p*<0.05); ** denotes Bonferroni-corrected significance (*p*<0.006 (0.05/9)).

Abbreviations: HbA1c, glycated hemoglobin

Table S3. Changes of FBG Responses in Q5 versus Q1 during metformin monotherapy by one or two clusters of risk alleles using East Asian specific- effect sizes

| Clusters | PRS group | Estimate | 95% CI | P-value |
| --- | --- | --- | --- | --- |
| Residual glycaemic | Q5 vs. Q1 | -0.227 | (-3.271, 2.818) | 0.884 |
| Obesity | Q5 vs. Q1 | 1.338 | (-1.795, 4.470) | 0.403 |
| **Beta cell +PI** | **Q5 vs. Q1** | **4.866** | **(1.990, 7.742)** | **0.0009**** |
| **Beta cell -PI** | **Q5 vs. Q1** | **5.929** | **(2.927, 8.931)** | **0.0001**** |
| Metabolic syndrome | Q5 vs. Q1 | -0.759 | (-3.687, 2.168) | 0.611 |
| Liver/lipid metabolism | Q5 vs. Q1 | 0.631 | (-1.883, 3.144) | 0.623 |
| Lipodystrophy | Q5 vs. Q1 | -0.201 | (-3.132, 2.730) | 0.893 |
| Body fat | Q5 vs. Q1 | -0.748 | (-3.722, 2.226) | 0.622 |
|  |  |  |  |  |
| **Beta cell +PI and Beta cell -PI** | **Both Q5 vs. Both Q1** | **9.545** | **(2.768, 16.322)** | **0.006**** |
| Beta cell +PI and Residual glycaemic | Both Q5 vs. Both Q1 | 3.903 | (-2.127, 9.933) | 0.205 |
| **Beta cell +PI and Obesity** | **Both Q5 vs. Both Q1** | **11.120** | **(4.680, 17.560)** | **0.001**** |
| Beta cell -PI and Residual glycaemic | Both Q5 vs. Both Q1 | 4.994 | (-1.989, 11.977) | 0.161 |
| Beta cell -PI and Obesity | Both Q5 vs. Both Q1 | 7.866 | (-0.115, 15.847) | 0.053 |
| Residual glycaemic and Obesity | Both Q5 vs. Both Q1 | 2.222 | (-3.859, 8.303) | 0.474 |

Abbreviations: FBG: Fasting blood glucose; Beta cell +PI or Beta cell -PI, beta-cell dysfunction with a positive or negative association with proinsulin, respectively.

* denotes nominal significance (*p*<0.05); ** denotes Bonferroni-corrected significance (*p*<0.006 (0.05/8 for single cluster) and p<0.008 (0.05/6, for both Q5 vs. both Q1)).

Table S4. Changes of FBG Responses in Q5 versus Q1 during metformin monotherapy by one or two clusters of risk alleles with further adjustment of BMI

| Clusters | PRS group | Estimate | 95% CI | P-value |
| --- | --- | --- | --- | --- |
| Residual glycaemic | Q5 vs. Q1 | 0.614 | (-3.054, 4.282) | 0.743 |
| Obesity | Q5 vs. Q1 | 2.967 | (-0.717, 6.652) | 0.114 |
| **Beta cell +PI** | **Q5 vs. Q1** | **6.512** | **(2.737, 10.286)** | **0.001**** |
| Beta cell -PI | Q5 vs. Q1 | 2.492 | (-1.186, 6.171) | 0.184 |
| Metabolic syndrome | Q5 vs. Q1 | -0.429 | (-3.938, 3.079) | 0.810 |
| **Liver/lipid metabolism** | **Q5 vs. Q1** | **3.600** | **(0.100, 7.100)** | **0.044*** |
| Lipodystrophy | Q5 vs. Q1 | 1.702 | (-1.848, 5.251) | 0.347 |
| Body fat | Q5 vs. Q1 | 0.437 | (-3.136, 4.010) | 0.811 |
|  |  |  |  |  |
| **Beta cell +PI and Beta cell -PI** | **Both Q5 vs. Both Q1** | **10.993** | **(1.738, 20.247)** | **0.020*** |
| Beta cell +PI and Residual glycaemic | Both Q5 vs. Both Q1 | 6.128 | (-1.131, 13.388) | 0.098 |
| **Beta cell +PI and Obesity** | **Both Q5 vs. Both Q1** | **14.842** | **(6.380, 23.303)** | **0.001**** |
| Beta cell -PI and Residual glycaemic | Both Q5 vs. Both Q1 | 0.838 | (-7.534, 9.210) | 0.845 |
| Beta cell -PI and Obesity | Both Q5 vs. Both Q1 | 2.787 | (-5.794, 11.369) | 0.524 |
| Residual glycaemic and Obesity | Both Q5 vs. Both Q1 | 1.974 | (-5.917, 9.865) | 0.624 |

Abbreviations: FBG: Fasting blood glucose; Beta cell +PI or Beta cell -PI, beta-cell dysfunction with a positive or negative association with proinsulin, respectively.

* denotes nominal significance (*p*<0.05); ** denotes Bonferroni-corrected significance (*p*<0.006 (0.05/8 for single cluster) and p<0.008 (0.05/6, for both Q5 vs. both Q1)).

Table S5. Changes of FBG Responses during metformin monotherapy by one or two clusters of risk alleles using continuous PRS

| Clusters | Estimate | 95% CI | P-value |
| --- | --- | --- | --- |
| Residual glycaemic | 0.539 | (-0.391, 1.468) | 0.256 |
| Obesity | 0.365 | (-0.583, 1.313) | 0.450 |
| **Beta cell +PI** | **1.143** | **(0.223, 2.064)** | **0.015*** |
| **Beta cell -PI** | **1.609** | **(0.656, 2.562)** | **0.001**** |
| Metabolic syndrome | -0.446 | (-1.379, 0.486) | 0.348 |
| Liver/lipid metabolism | 0.281 | (-0.653, 1.215) | 0.555 |
| Lipodystrophy | 0.189 | (-0.747, 1.126) | 0.692 |
| Body fat | 0.062 | (-0.864, 0.988) | 0.896 |
|  |  |  |  |
| **Beta cell +PI and Beta cell -PI** | **1.353** | **(0.681, 2.025)** | **0.00008**** |
| **Beta cell +PI and Residual glycaemic** | **0.784** | **(0.145, 1.424)** | **0.016*** |
| **Beta cell +PI and Obesity** | **0.768** | **(0.112, 1.425)** | **0.022*** |
| **Beta cell -PI and Residual glycaemic** | **1.057** | **(0.372, 1.741)** | **0.002**** |
| **Beta cell -PI and Obesity** | **1.003** | **(0.309, 1.697)** | **0.005**** |
| Residual glycaemic and Obesity | 0.470 | (-0.170, 1.109) | 0.150 |

Abbreviations: FBG: Fasting blood glucose; Beta cell +PI or Beta cell -PI, beta-cell dysfunction with a positive or negative association with proinsulin, respectively.

* denotes nominal significance (*p*<0.05); ** denotes Bonferroni-corrected significance (*p*<0.006 (0.05/8 for single cluster) and p<0.008 (0.05/6, for both Q5 vs. both Q1)).

Table S6. Changes of HbA1c Responses in Q5 versus Q1 during metformin monotherapy by one or two clusters of risk alleles using East Asian specific- effect sizes

| Clusters | PRS group | Estimate | 95% CI | P-value |
| --- | --- | --- | --- | --- |
| **Residual glycaemic** | **Q5 vs. Q1** | **-0.256** | **(-0.331, -0.180)** | **3.66E-11**** |
| Obesity | Q5 vs. Q1 | 0.018 | (-0.104, 0.139) | 0.774 |
| Beta cell +PI | Q5 vs. Q1 | -0.063 | (-0.176, 0.050) | 0.272 |
| **Beta cell -PI** | **Q5 vs. Q1** | **0.203** | **(0.094, 0.312)** | **0.0003**** |
| Metabolic syndrome | Q5 vs. Q1 | -0.004 | (-0.120, 0.111) | 0.941 |
| Liver/lipid metabolism | Q5 vs. Q1 | 0.043 | (-0.063, 0.150) | 0.424 |
| Lipodystrophy | Q5 vs. Q1 | 0.013 | (-0.100, 0.126) | 0.820 |
| Body fat | Q5 vs. Q1 | 0.058 | (-0.052, 0.167) | 0.303 |
|  |  |  |  |  |
| Beta cell -PI and Residual glycaemic | Both Q5 vs. Both Q1 | 0.043 | (-0.195, 0.280) | 0.725 |
| Beta cell -PI and Obesity | Both Q5 vs. Both Q1 | 0.214 | (-0.075, 0.502) | 0.147 |
| Beta cell -PI and Beta cell +PI | Both Q5 vs. Both Q1 | 0.024 | (-0.215, 0.264) | 0.842 |
| Beta cell +PI and Residual glycaemic | Both Q5 vs. Both Q1 | -0.177 | (-0.361, 0.008) | 0.060 |
| Beta cell +PI and Obesity | Both Q5 vs. Both Q1 | 0.096 | (-0.210, 0.401) | 0.539 |
| Residual glycaemic and Obesity | Both Q5 vs. Both Q1 | -0.066 | (-0.345, 0.213) | 0.641 |

Abbreviations: HbA1c, glycated hemoglobin; Beta cell +PI or Beta cell -PI, beta-cell dysfunction with a positive or negative association with proinsulin, respectively.

* denotes nominal significance (*p*<0.05); ** denotes Bonferroni-corrected significance (*p*<0.006 (0.05/8 for single cluster) and p<0.008 (0.05/6, for both Q5 vs. both Q1)).

Table S7. Changes of HbA1c Responses in Q5 versus Q1 during metformin monotherapy by one or two clusters of risk alleles with further adjustment of BMI

| Clusters | PRS group | Estimate | 95% CI | P-value |
| --- | --- | --- | --- | --- |
| Residual glycaemic | Q5 vs. Q1 | 0.102 | (-0.035, 0.238) | 0.143 |
| Obesity | Q5 vs. Q1 | -0.009 | (-0.158, 0.141) | 0.909 |
| Beta cell +PI | Q5 vs. Q1 | -0.087 | (-0.226, 0.053) | 0.223 |
| **Beta cell -PI** | **Q5 vs. Q1** | **0.164** | **(0.030, 0.299)** | **0.017*** |
| Metabolic syndrome | Q5 vs. Q1 | 0.003 | (-0.151, 0.157) | 0.967 |
| Liver/lipid metabolism | Q5 vs. Q1 | 0.086 | (-0.066, 0.238) | 0.266 |
| Lipodystrophy | Q5 vs. Q1 | 0.064 | (-0.070, 0.197) | 0.350 |
| Body fat | Q5 vs. Q1 | 0.088 | (-0.043, 0.218) | 0.187 |
|  |  |  |  |  |
| Beta cell -PI and Residual glycaemic | Both Q5 vs. Both Q1 | 0.186 | (-0.136, 0.508) | 0.257 |
| Beta cell -PI and Obesity | Both Q5 vs. Both Q1 | 0.010 | (-0.386, 0.406) | 0.961 |
| Beta cell -PI and Beta cell +PI | Both Q5 vs. Both Q1 | 0.068 | (-0.259, 0.394) | 0.685 |
| Beta cell +PI and Residual glycaemic | Both Q5 vs. Both Q1 | 0.044 | (-0.310, 0.399) | 0.806 |
| Beta cell +PI and Obesity | Both Q5 vs. Both Q1 | 0.023 | (-0.365, 0.411) | 0.908 |
| Residual glycaemic and Obesity | Both Q5 vs. Both Q1 | 0.115 | (-0.287, 0.517) | 0.577 |

Abbreviations: HbA1c, glycated hemoglobin; Beta cell +PI or Beta cell -PI, beta-cell dysfunction with a positive or negative association with proinsulin, respectively.

* denotes nominal significance (*p*<0.05); ** denotes Bonferroni-corrected significance (*p*<0.006 (0.05/8 for single cluster) and p<0.008 (0.05/6, for both Q5 vs. both Q1)).

Table S8. Changes of HbA1c Responses during metformin monotherapy by one or two clusters of risk alleles using continuous PRS

| Clusters | Estimate | 95% CI | P-value |
| --- | --- | --- | --- |
| Residual glycaemic | -0.004 | (-0.040, 0.033) | 0.848 |
| Obesity | 0.003 | (-0.036, 0.042) | 0.877 |
| **Beta cell +PI** | **-0.043** | **(-0.081, -0.004)** | **0.030*** |
| **Beta cell -PI** | **0.060** | **(0.021, 0.099)** | **0.003**** |
| Metabolic syndrome | 0.004 | (-0.034, 0.042) | 0.829 |
| Liver/lipid metabolism | -0.013 | (-0.050, 0.025) | 0.514 |
| Lipodystrophy | 0.014 | (-0.025, 0.053) | 0.490 |
| Body fat | 0.025 | (-0.013, 0.063) | 0.189 |
|  |  |  |  |
| **Beta cell -PI and Residual glycaemic** | **0.028** | **(0.001, 0.055)** | **0.042*** |
| **Beta cell -PI and Obesity** | **0.032** | **(0.003, 0.062)** | **0.030*** |
| Beta cell -PI and Beta cell +PI | 0.009 | (-0.019, 0.036) | 0.534 |
| Beta cell +PI and Residual glycaemic | -0.022 | (-0.048, 0.004) | 0.102 |
| Beta cell +PI and Obesity | -0.020 | (-0.048, 0.008) | 0.153 |
| Residual glycaemic and Obesity | -0.0002 | (-0.026, 0.025) | 0.986 |

Abbreviations: HbA1c, glycated hemoglobin; Beta cell +PI or Beta cell -PI, beta-cell dysfunction with a positive or negative association with proinsulin, respectively.

* denotes nominal significance (*p*<0.05); ** denotes Bonferroni-corrected significance (*p*<0.006 (0.05/8 for single cluster) and p<0.008 (0.05/6, for both Q5 vs. both Q1)).

Table S9. Clinical characteristics of individuals with type 2 diabetes carrying Q5 versus Q1 of PRS of Beta cell +PI

| Characteristics | Type 2 Diabetes carry Q1 of PRS  (N=512) | Type 2 Diabetes carry Q5 of PRS  (N=577) | p-value |
| --- | --- | --- | --- |
| **Age (years)** | **59.9 ± 10.9** | **57.5 ± 12.0** | **0.004**** |
| Female, n (%) | 217 (42.4) | 242 (41.9) | 0.883 |
| **BMI (kg/m^2^)** | **26.9 ± 4.0** | **26.0 ± 4.0** | **0.003**** |
| Baseline Fasting Glucose (mg/dl) | 137.2 ± 27.7 | 140.1 ± 27.4 | 0.175 |
| Change in Fasting Glucose on metformin treatment | -14.6 ± 28.3 | -12.8 ± 28.4 | 0.538 |
| Baseline HbA1c (%) | 8.0 ± 1.9 | 7.8 ± 1.6 | 0.451 |
| Change in HbA1c on metformin treatment | -1.1 ± 1.9 | -1.0 ± 1.5 | 0.986 |
| **Metformin use (mg/day)** | **536 ± 207** | **559 ± 218** | **0.030*** |
| Duration of metformin use (month) | 4.8 ± 1.3 | 4.8 ± 1.4 | 0.941 |

Data are presented as either the number (percentage) or as the mean ± standard deviation. The Kruskal-Wallis test or$\chi_{2}^{2}$test was conducted to evaluate the differences between two groups.

* denotes nominal significance (*p*<0.05); ** denotes Bonferroni-corrected significance (*p*<0.006 (0.05/9)).

Abbreviations: HbA1c, glycated hemoglobin; Beta cell +PI, beta-cell dysfunction with a positive association with proinsulin.

Table S10. Clinical characteristics of individuals with type 2 diabetes carrying Q5 versus Q1 of PRS of Beta cell -PI

| Characteristics | Type 2 Diabetes carry Q1 of PRS  (N=532) | Type 2 Diabetes carry Q5 of PRS  (N=554) | p-value |
| --- | --- | --- | --- |
| Age (years) | 59.3 ± 11.4 | 58.1 ± 11.7 | 0.138 |
| Female, n (%) | 227 (42.7) | 249 (45.0) | 0.450 |
| BMI (kg/m^2^) | 26.6 ± 3.7 | 26.2 ± 4.0 | 0.123 |
| Baseline Fasting Glucose (mg/dl) | 137.7 ± 27.5 | 137.0 ± 26.7 | 0.896 |
| Change in Fasting Glucose on metformin treatment | -12.8 ± 28.8 | -9.0 ± 25.0 | 0.345 |
| Baseline HbA1c (%) | 7.7 ± 1.5 | 7.9 ± 1.9 | 0.392 |
| Change in HbA1c on metformin treatment | -1.0 ± 1.4 | -1.0 ± 1.8 | 0.631 |
| **Metformin use (mg/day)** | **554 ± 242** | **565 ± 215** | **0.018*** |
| Duration of metformin use (month) | 4.8 ± 1.4 | 4.8 ± 1.3 | 0.561 |

Data are presented as either the number (percentage) or as the mean ± standard deviation. The Kruskal-Wallis test or$\chi_{2}^{2}$test was conducted to evaluate the differences between two groups.

* denotes nominal significance (*p*<0.05); ** denotes Bonferroni-corrected significance (*p*<0.006 (0.05/9)).

Abbreviations: HbA1c, glycated hemoglobin; Beta cell -PI, beta-cell dysfunction with a negative association with proinsulin.

Table S11. Clinical characteristics of individuals with type 2 diabetes carrying Q5 versus Q1 of PRS of residual glycaemic

| Characteristics | Type 2 Diabetes carry Q1 of PRS  (N=515) | Type 2 Diabetes carry Q5 of PRS  (N=545) | p-value |
| --- | --- | --- | --- |
| **Age (years)** | **60.2 ± 11.5** | **57.6 ± 12.1** | **0.0004**** |
| Female, n (%) | 212 (41.2) | 239 (43.9) | 0.376 |
| **BMI (kg/m^2^)** | **26.7 ± 3.8** | **25.9 ± 3.9** | **0.007*** |
| Baseline Fasting Glucose (mg/dl) | 136.3 ± 29.0 | 138.8 ± 25.4 | 0.175 |
| Change in Fasting Glucose on metformin treatment | -11.3 ± 27.5 | -12.6 ± 26.4 | 0.423 |
| Baseline HbA1c (%) | 7.8 ± 1.7 | 7.8 ± 1.8 | 0.408 |
| Change in HbA1c on metformin treatment | -1.1 ± 1.7 | -1.0 ± 1.7 | 0.645 |
| Metformin use (mg/day) | 555 ± 234 | 539 ± 193 | 0.381 |
| Duration of metformin use (month) | 4.8 ± 1.3 | 4.8 ± 1.3 | 0.940 |

Data are presented as either the number (percentage) or as the mean ± standard deviation. The Kruskal-Wallis test or$\chi_{2}^{2}$test was conducted to evaluate the differences between two groups.

* denotes nominal significance (*p*<0.05); ** denotes Bonferroni-corrected significance (*p*<0.006 (0.05/9)).

Abbreviations: HbA1c, glycated hemoglobin.

Table S12. Clinical characteristics of individuals with type 2 diabetes carrying Q5 versus Q1 of PRS of obesity

| Characteristics | Type 2 Diabetes carry Q1 of PRS  (N=559) | Type 2 Diabetes carry Q5 of PRS  (N=520) | p-value |
| --- | --- | --- | --- |
| **Age (years)** | **60.2 ± 12.0** | **58.2 ± 12.2** | **0.004**** |
| Female, n (%) | 239 (42.8) | 252 (48.5) | 0.060 |
| **BMI (kg/m^2^)** | **26.1 ± 3.9** | **27.3 ± 3.8** | **0.0001**** |
| Baseline Fasting Glucose (mg/dl) | 139.3 ± 28.5 | 138.3 ± 25.4 | 0.838 |
| Change in Fasting Glucose on metformin treatment | -13.2 ± 28.4 | -11.9 ± 26.1 | 0.991 |
| Baseline HbA1c (%) | 7.8 ± 1.7 | 7.9 ± 1.8 | 0.807 |
| Change in HbA1c on metformin treatment | -1.0 ± 1.6 | -1.1 ± 1.9 | 0.958 |
| Metformin use (mg/day) | 546 ± 218 | 551 ± 209 | 0.436 |
| Duration of metformin use (month) | 4.9 ± 1.4 | 4.7 ± 1.4 | 0.249 |

Data are presented as either the number (percentage) or as the mean ± standard deviation. The Kruskal-Wallis test or$\chi_{2}^{2}$test was conducted to evaluate the differences between two groups.

* denotes nominal significance (*p*<0.05); ** denotes Bonferroni-corrected significance (*p*<0.006 (0.05/9)).

Abbreviations: HbA1c, glycated hemoglobin.

Fig. S1 Trends of fasting glucose responses to metformin monotherapy of Q5 vs. Q1 in each cluster not shown in Figure 2.


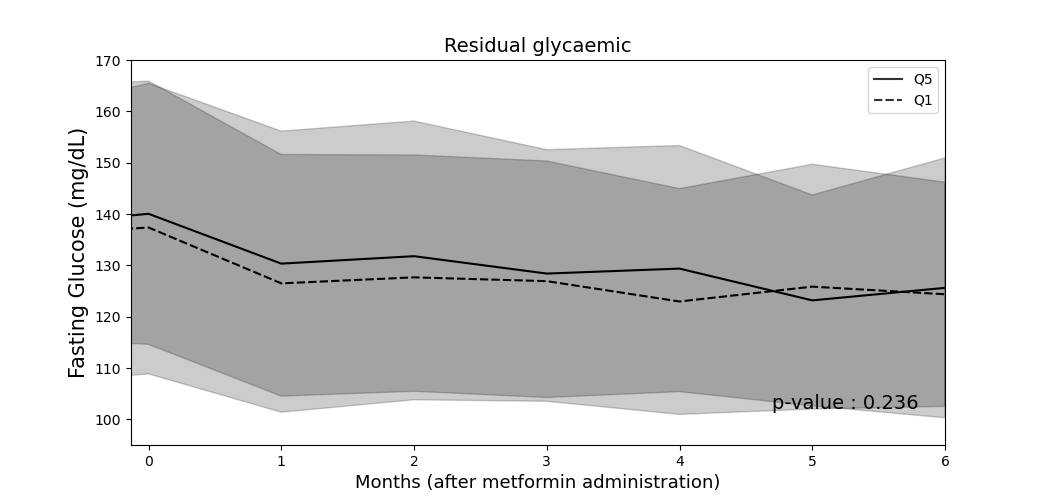

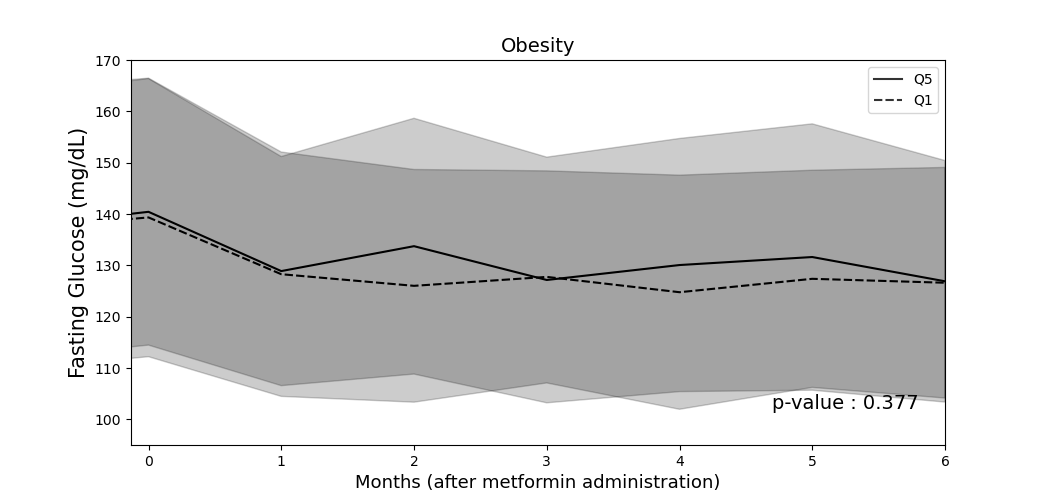

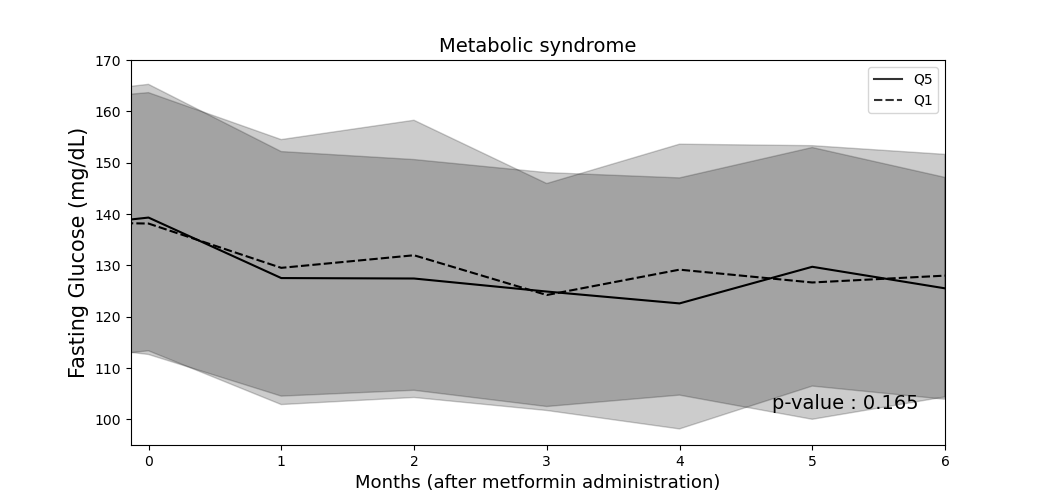


A

B

C


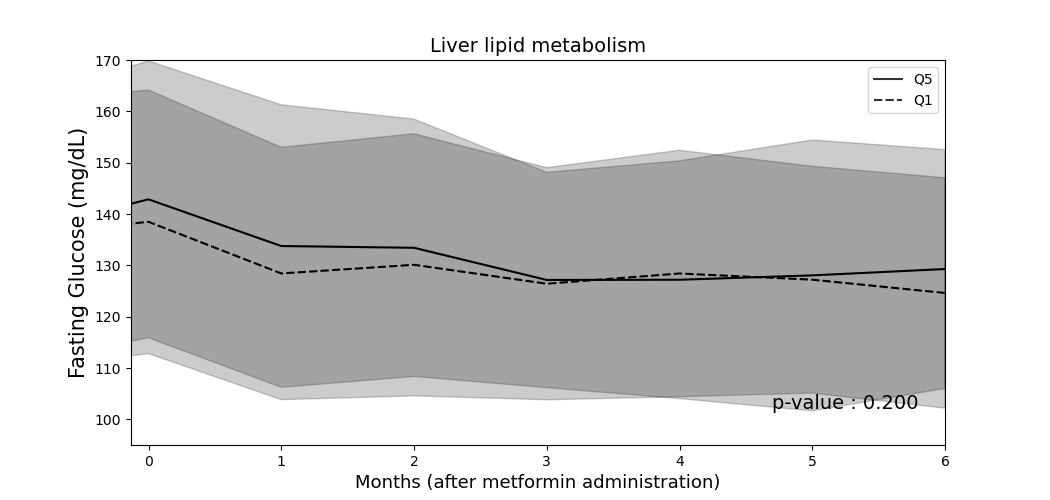

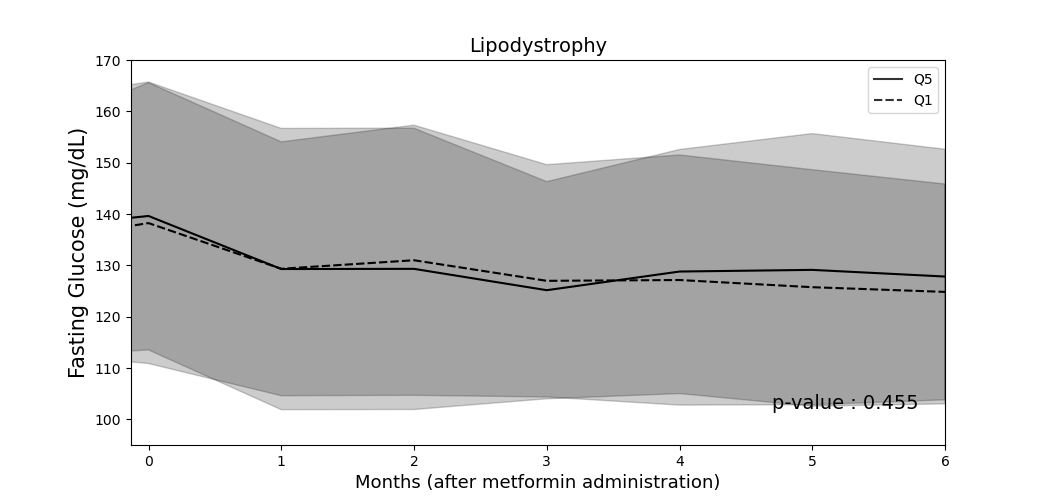

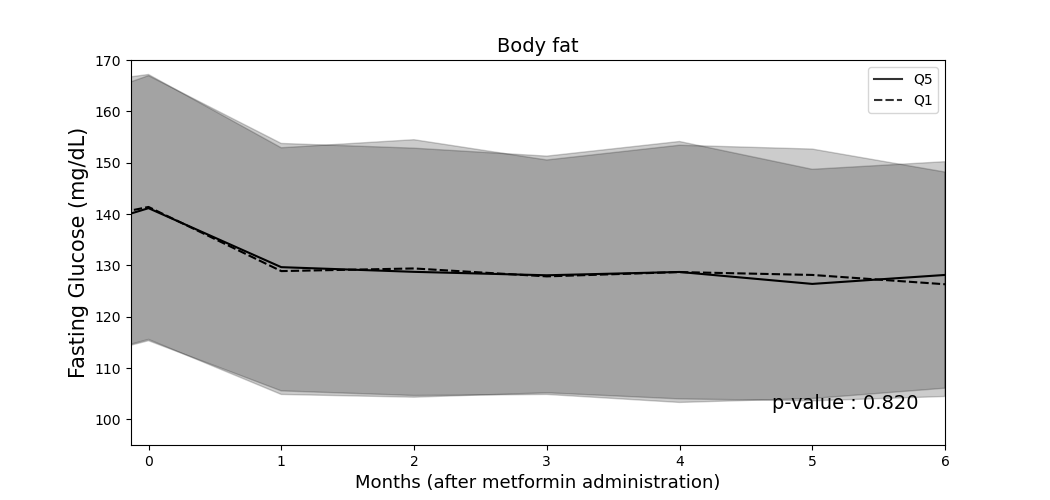


D

E

F

Fig. S2 Trends of FBG responses to metformin monotherapy of Q5 vs. Q1 in those two clusters not shown in Figure 2.


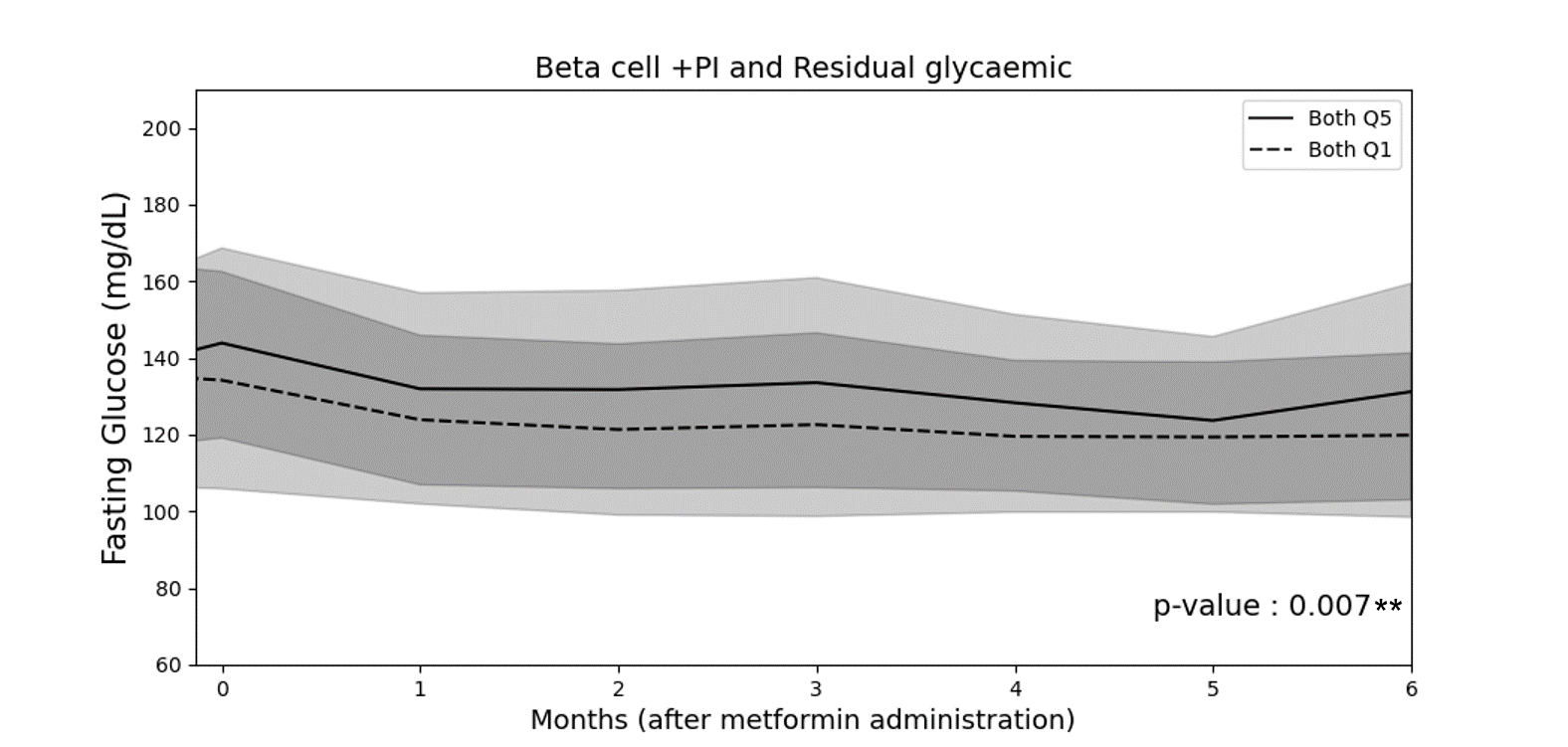

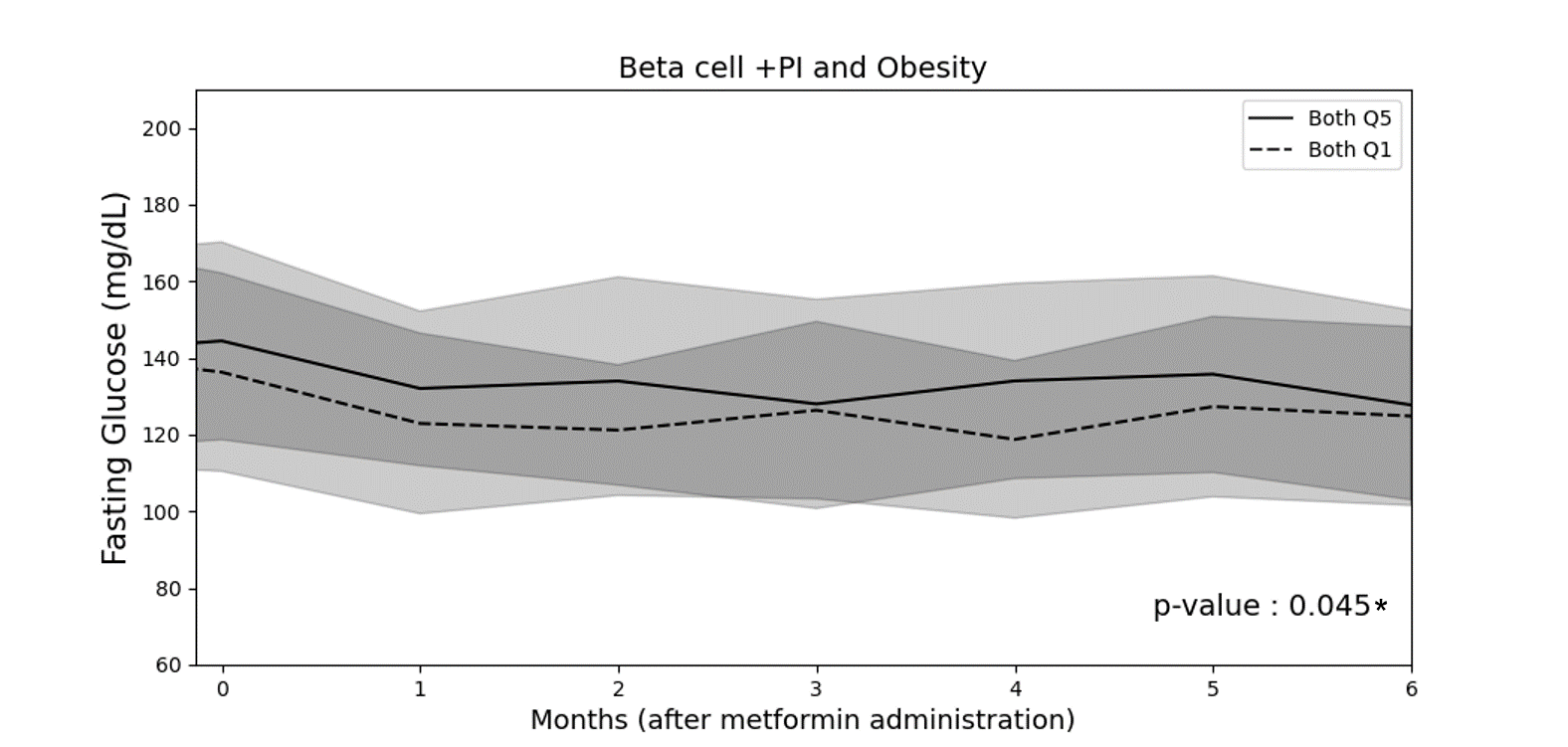

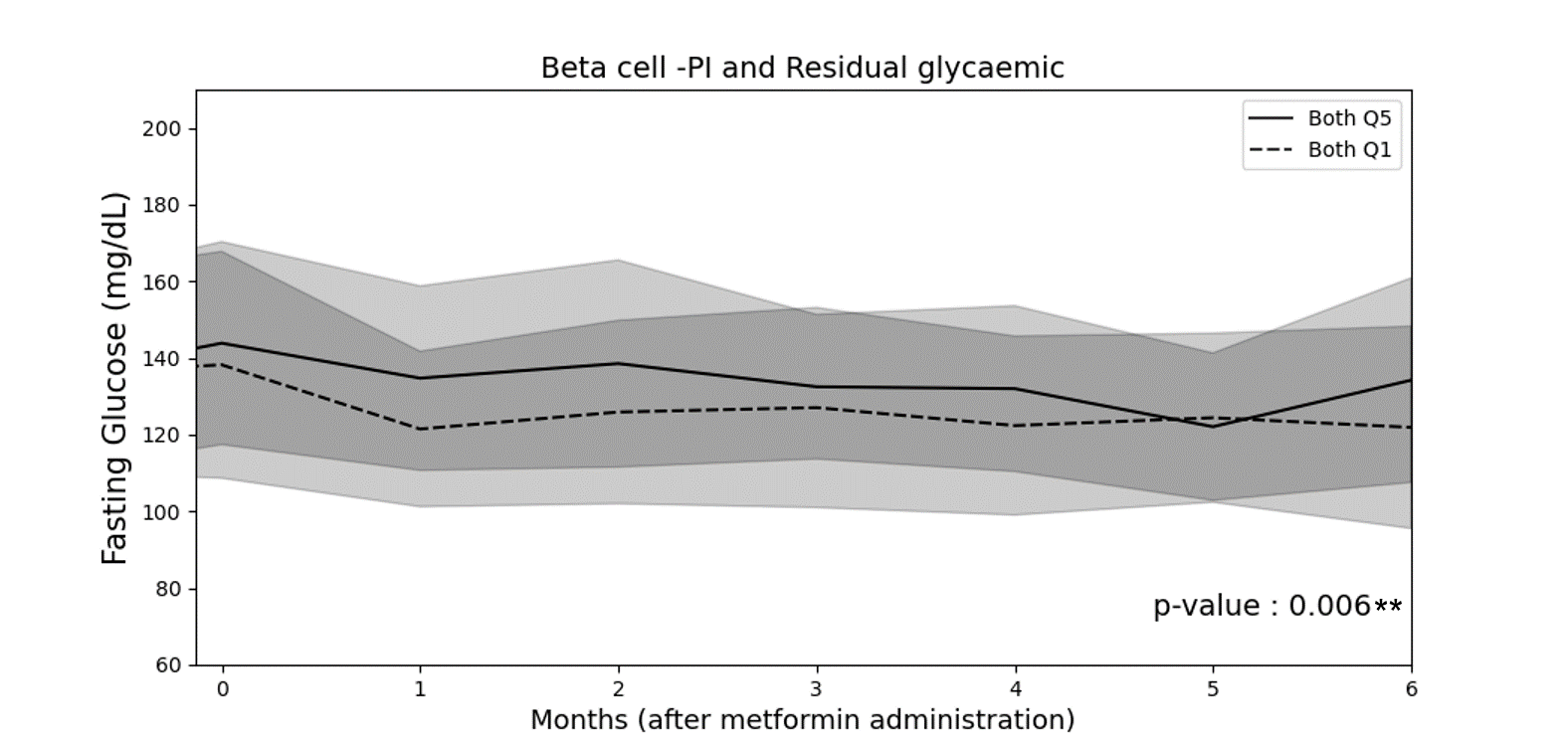


A

B

C


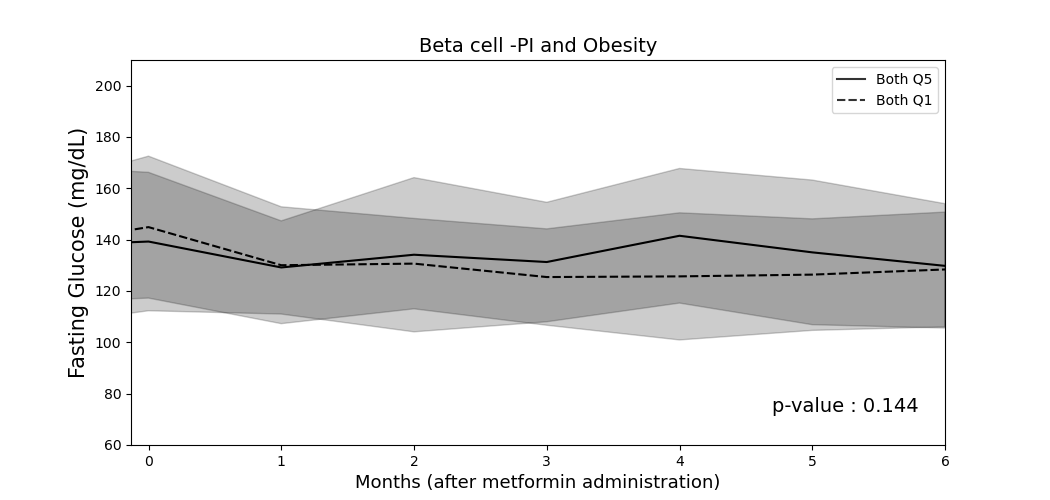

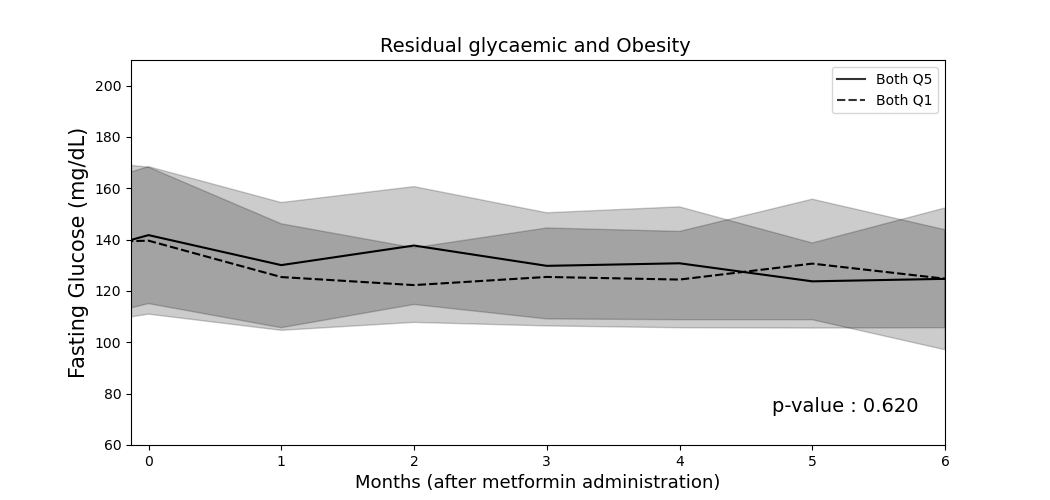


D

E

Fig. S3 Trends of HbA1c responses to metformin monotherapy of Q5 vs. Q1 in Beta cell -PI, residual glycaemic and combination of two clusters.


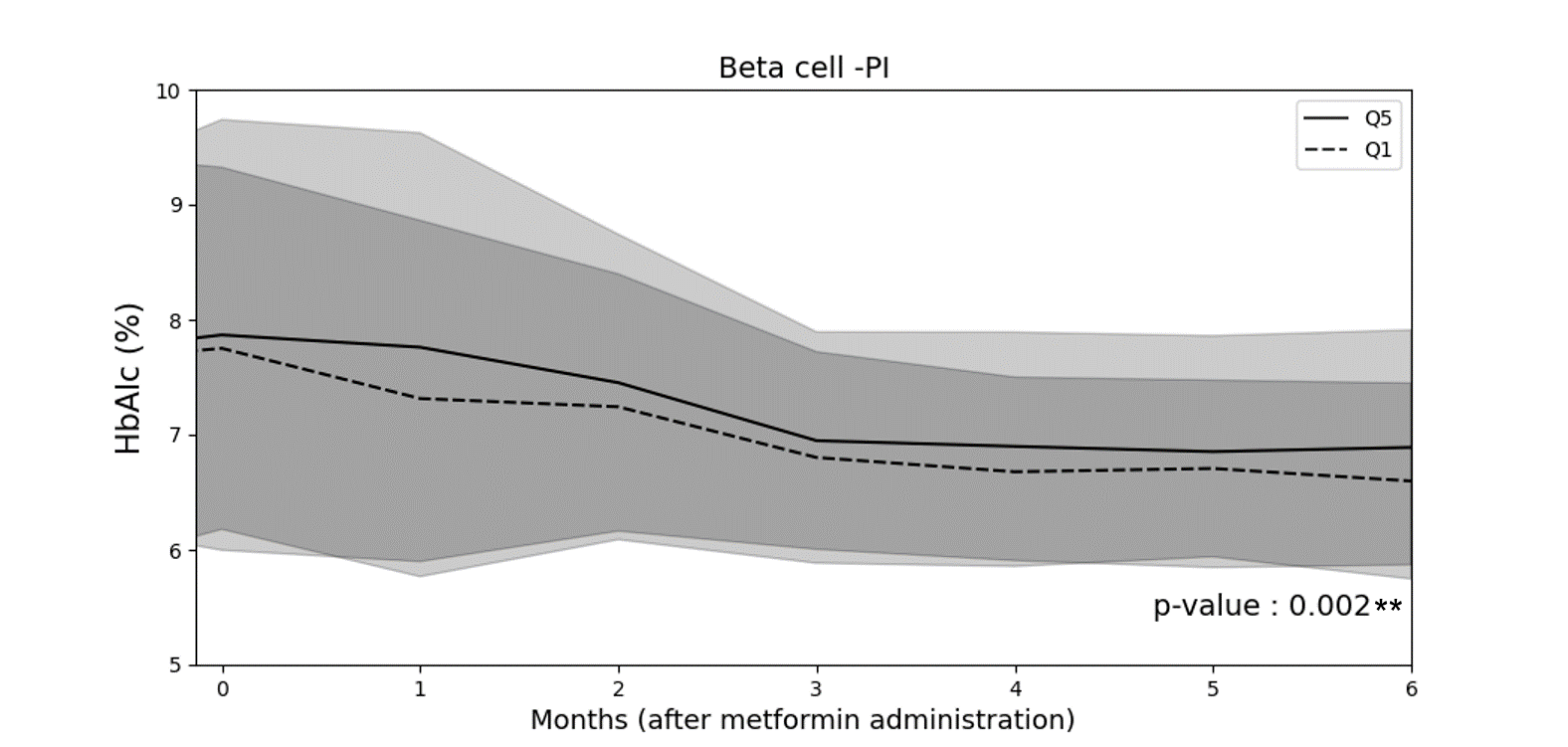

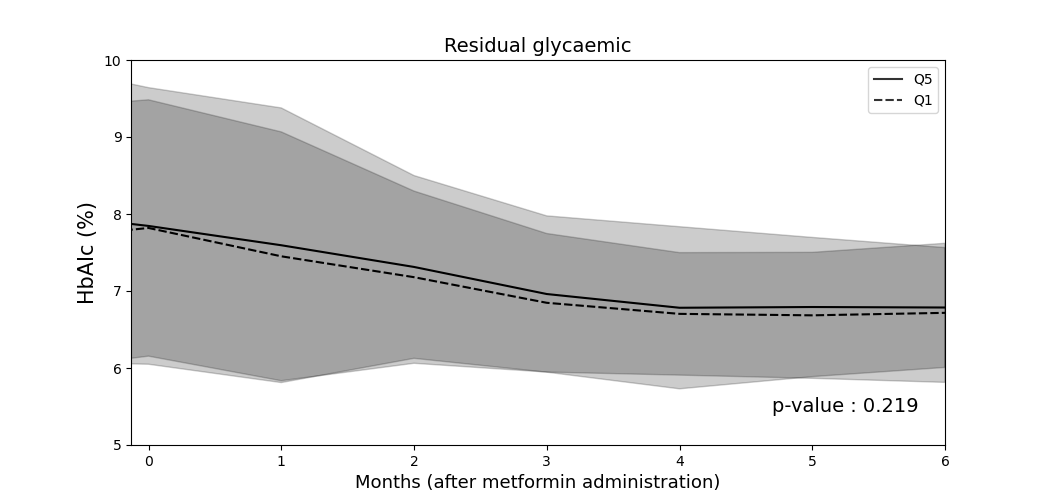

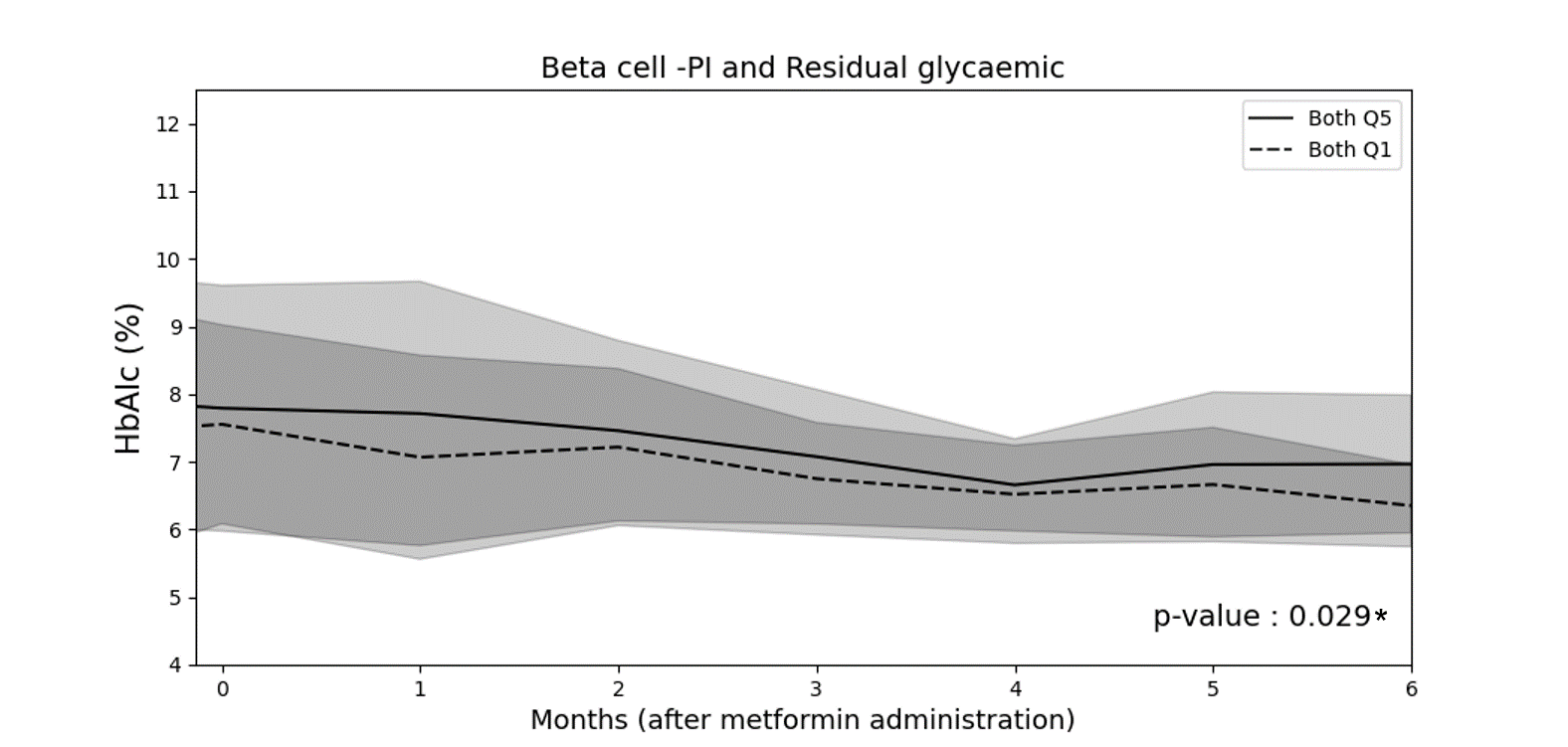


A.

B.

C.

Fig. S4 Trends of HbA1c responses to metformin monotherapy of Q5 vs. Q1 in each cluster not shown in Figure S3.


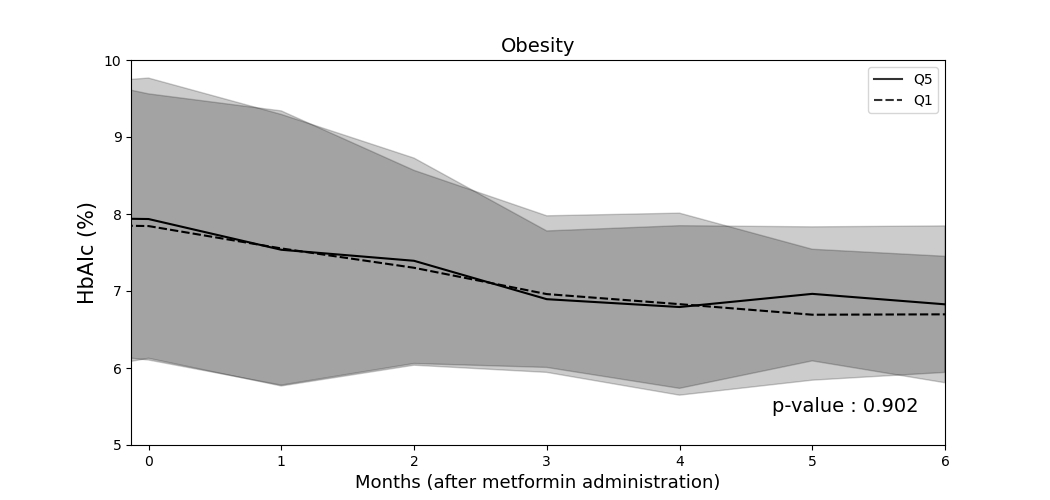

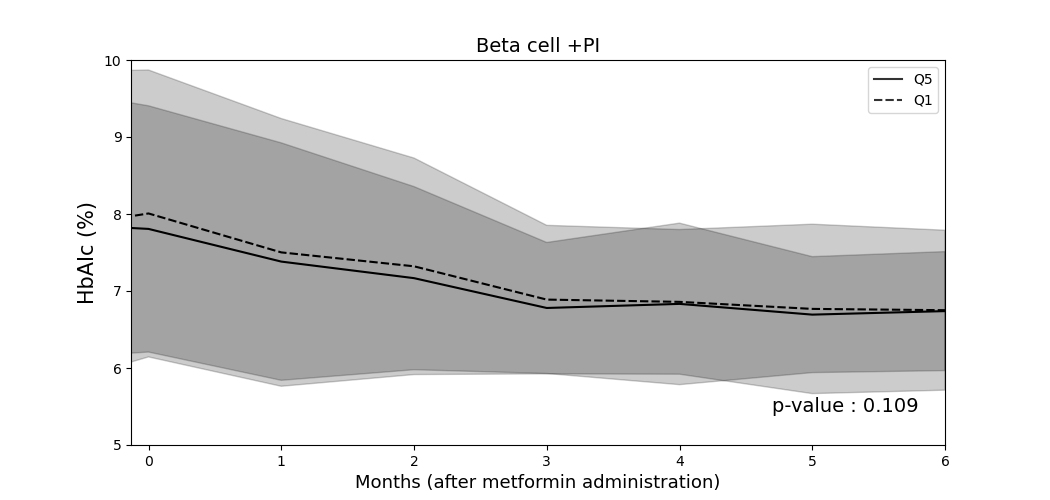

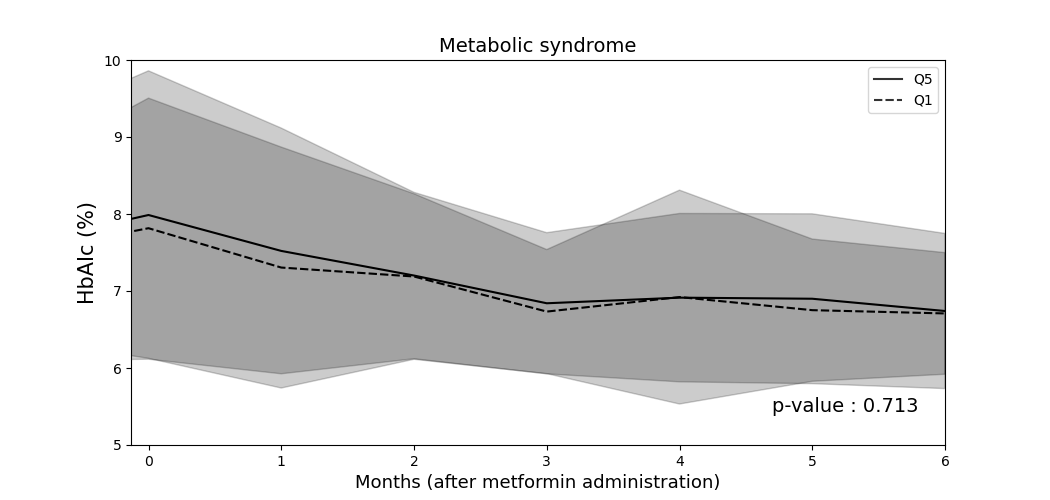


A

B

C


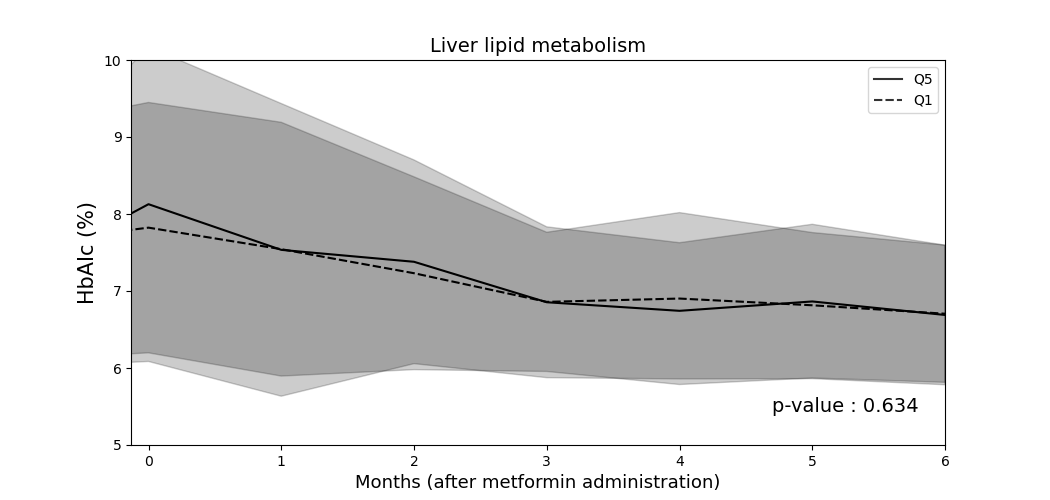

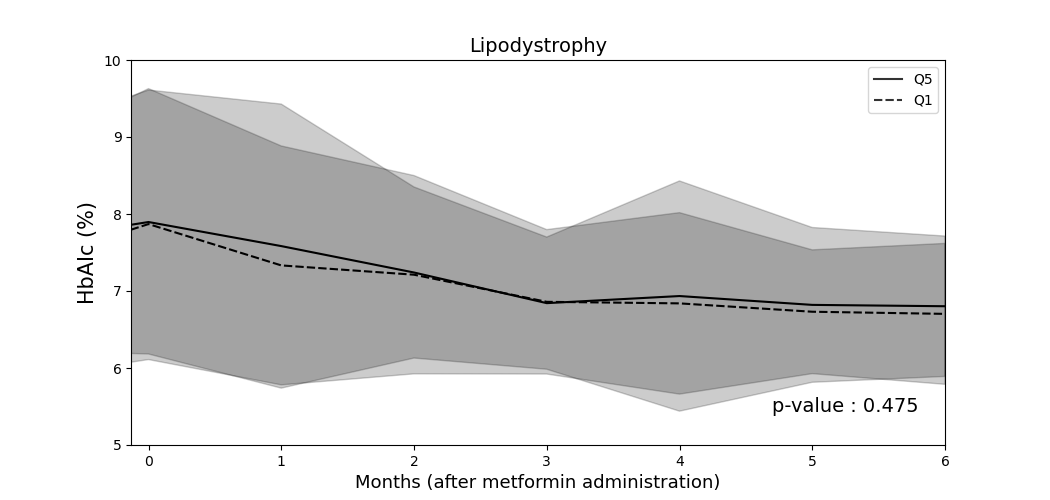

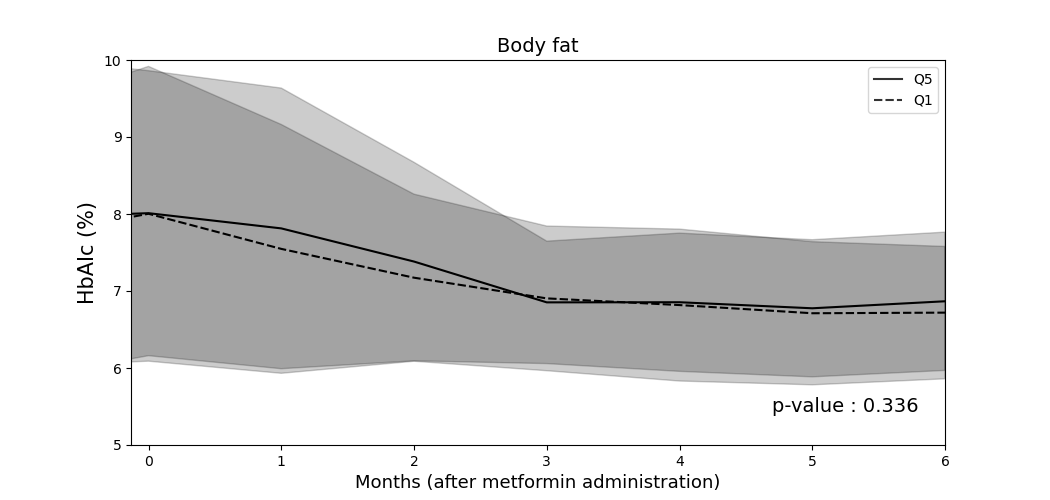


D

E

F

Fig. S5 Trends of HbA1c responses to metformin monotherapy of Q5 vs. Q1 in those two clusters not shown in Figure S3.


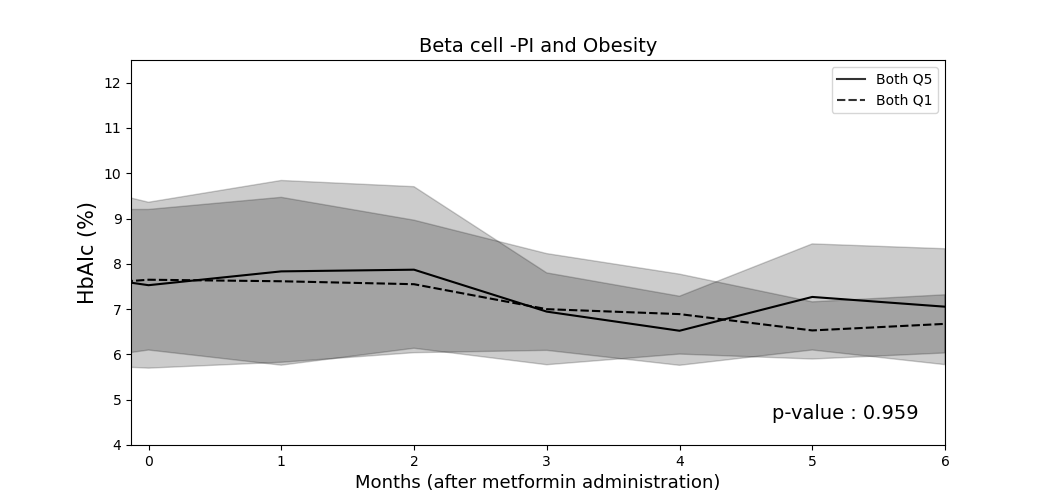

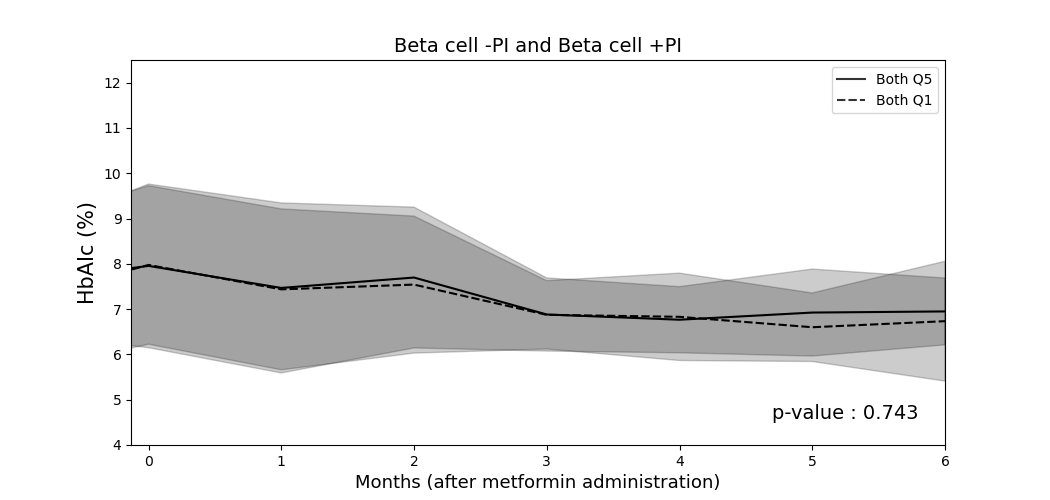

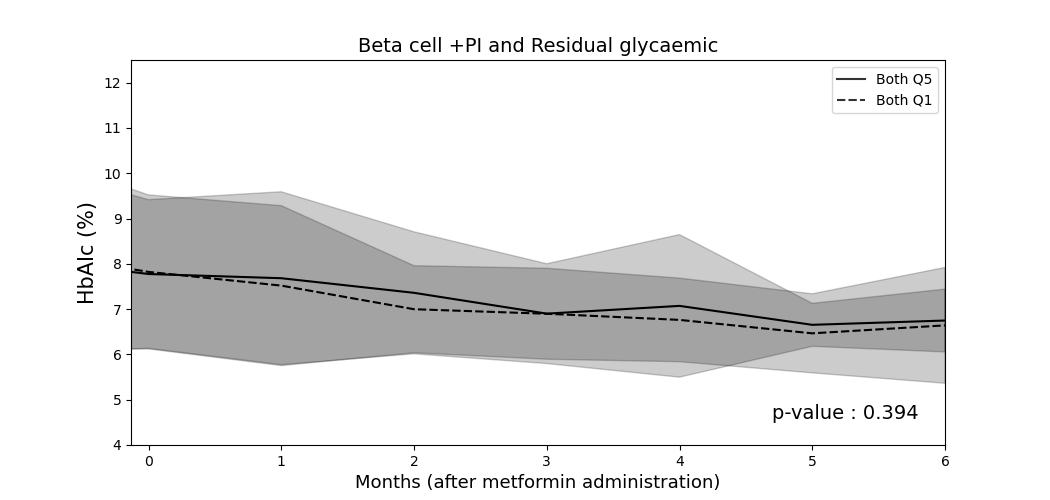


A

B

C


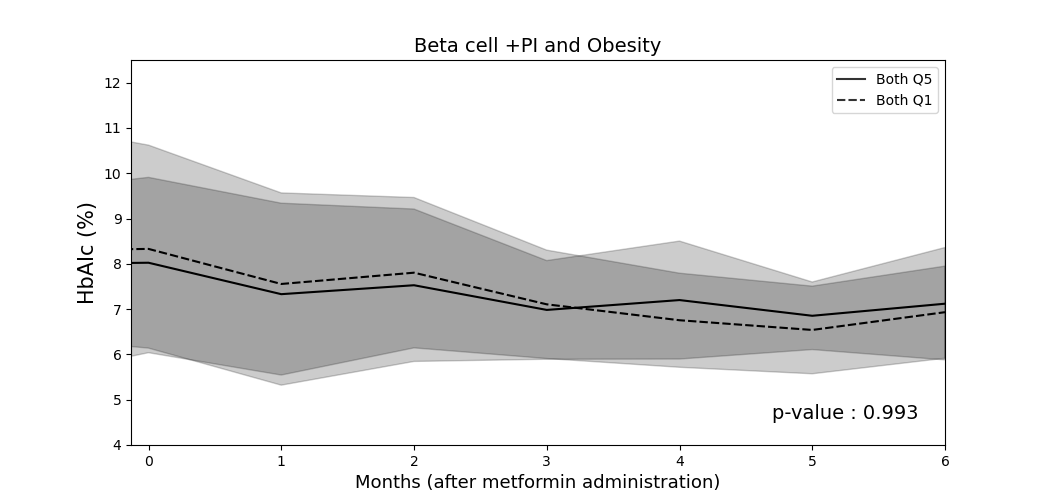

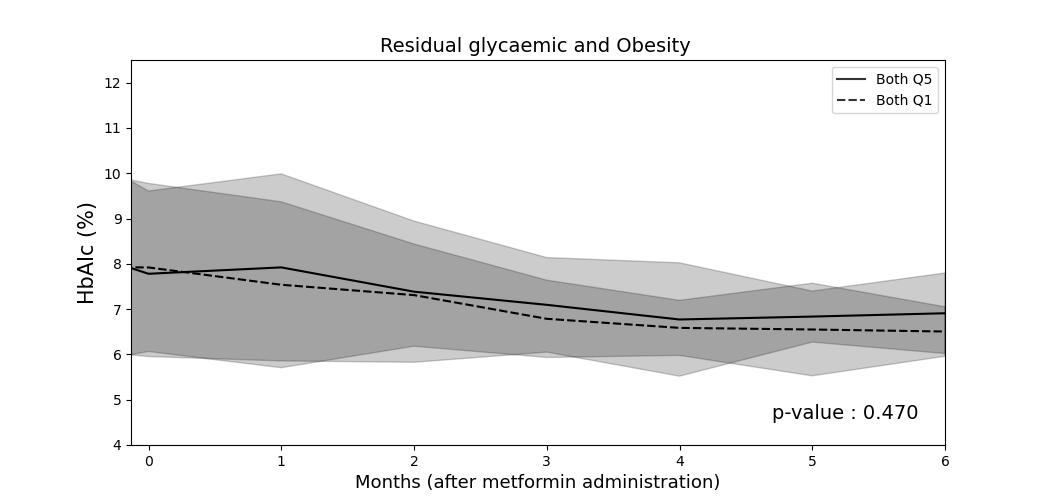


D

E

Fig. S6 Change of FBG/HbA1c responses across PRS spectrum during metformin monotherapy.


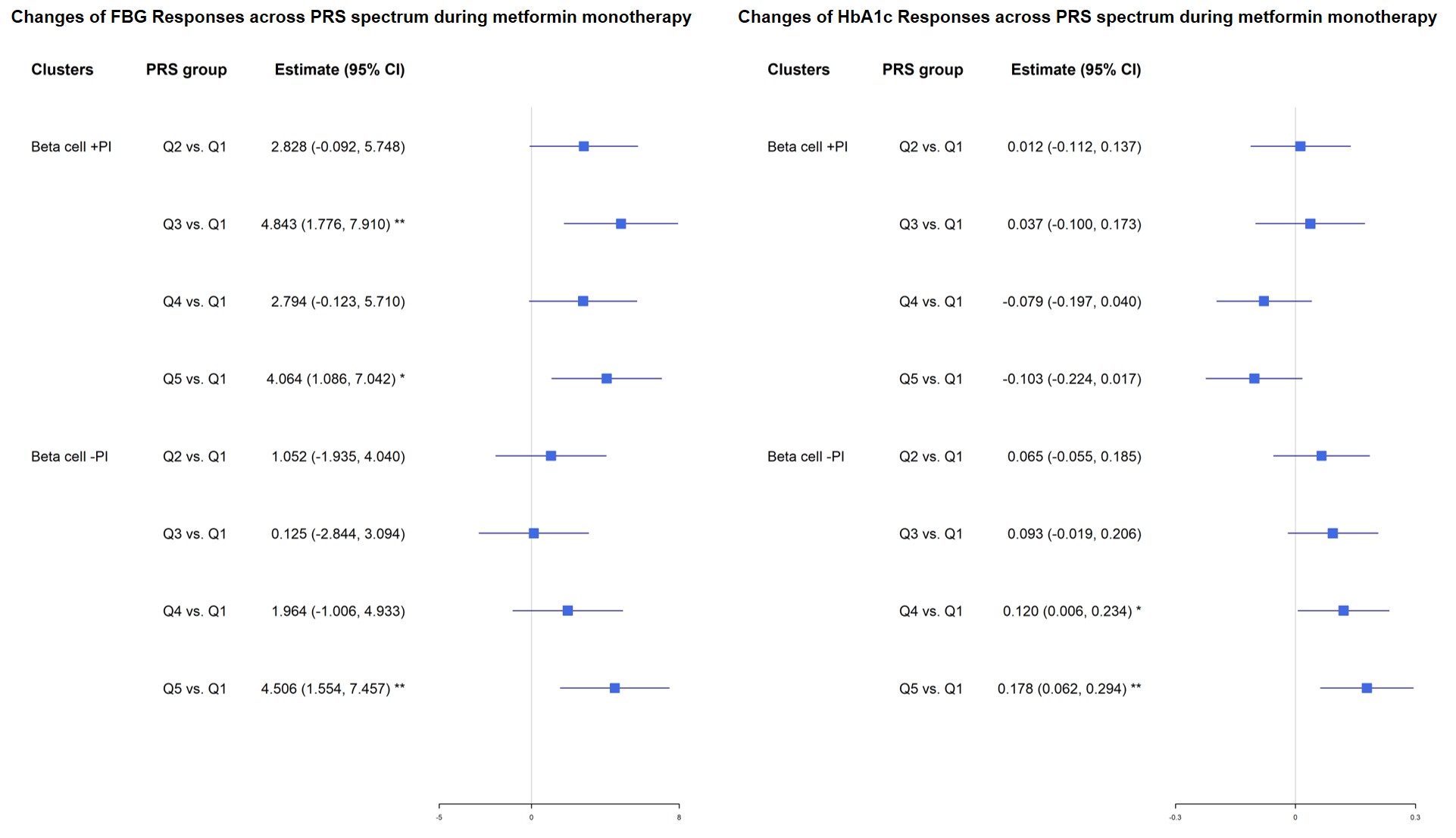

Supplement: Supplementary file 1 — Data S1. Supporting Information. [file DOM-27-6323-s001.docx]
